# Identification of C/EBPβ Target Genes in ALK+ Anaplastic Large Cell Lymphoma (ALCL) by Gene Expression Profiling and Chromatin Immunoprecipitation

**DOI:** 10.1371/journal.pone.0064544

**Published:** 2013-05-31

**Authors:** Irina Bonzheim, Martin Irmler, Margit Klier-Richter, Julia Steinhilber, Nataša Anastasov, Sabine Schäfer, Patrick Adam, Johannes Beckers, Mark Raffeld, Falko Fend, Leticia Quintanilla-Martinez

**Affiliations:** 1 Institute of Pathology and Neuropathology, University Hospital Tübingen and Comprehensive Cancer Center, Eberhard-Karls-University, Tübingen, Germany; 2 Institute of Pathology, Helmholtz Zentrum München GmbH - German Research Center for Environmental Health, Neuherberg, Germany; 3 Institute of Experimental Genetics, Helmholtz Zentrum München GmbH - German Research Center for Environmental Health, Neuherberg, Germany; 4 National Cancer Institute, National Institutes of Health, Bethesda, Maryland, United States of America; 5 Chair of Experimental Genetics, Technische Universität München, Freising-Weihenstephan, Germany; University of Navarra, Center for Applied Medical Research, Spain

## Abstract

C/EBPβ (CCAAT enhancer binding protein) is a transcription factor that plays a crucial role in survival and transformation of ALK+ anaplastic large cell lymphoma (ALCL). The aim of this study was to identify the downstream targets of C/EBPβ responsible for ALK-mediated oncogenesis. *C/EBPβ* was knocked down in ALK+ ALCL cell lines with a C/EBPβ-shRNA, followed by gene expression profiling (GEP). GEP analysis revealed a reproducible signature of genes that were significantly regulated by C/EBPβ. Classification into biological categories revealed overrepresentation of genes involved in the immune response, apoptosis and cell proliferation. Transcriptional regulation by C/EBPβ was found in 6 of 11 (*BCL2A1, G0S2, TRIB1, S100A9, DDX21 and DDIT4*) genes investigated by chromatin immunoprecipitation. We demonstrated that *BCL2A1*, *G0S2 and DDX21* play a crucial role in survival and proliferation of ALK+ ALCL cells. *DDX21*, a gene involved in rRNA biogenesis, was found differentially overexpressed in primary ALK+ ALCL cases. All three candidate genes were validated in primary ALCL cases by either immunohistochemistry or RT-qPCR. In conclusion, we identified and validated several key C/EBPβ-regulated genes with major impact on survival and cell growth in ALK+ ALCL, supporting the central role of C/EBPβ in ALK-mediated oncogenesis.

## Introduction

The transcription factor CCAAT/enhancer binding protein beta (C/EBPβ) is one of six members of the C/EBP leucine zipper transcription factor family. *C/EBPβ* is an intronless gene, which is transcribed as a single mRNA that can produce at least three isoforms (liver-enriched activating proteins LAP (46 kDa) and LAP* (48 kDa) and liver-enriched inhibitory protein LIP (21 kDa)). It is involved in a number of cellular processes, including differentiation, proliferation, inflammatory responses and metabolism [1], [2]. Furthermore, C/EBPβ has been implicated in oncogene-mediated tumorigenesis and apoptosis resistance in solid tumors [3], [4].

Recently, we reported that C/EBPβ is overexpressed in anaplastic lymphoma kinase (ALK)+anaplastic large cell lymphoma (ALCL), and demonstrated that its expression is dependent on ALK kinase activity [5], [6]. ALK+ ALCL is a distinct subtype of non-Hodgkin’s lymphoma with unique morphologic and immunophenotypical features. ALK+ ALCL is characterized by the t(2;5) chromosomal translocation, which juxtaposes the nucleophosmin (*NPM*) gene and the *ALK* gene, resulting in the expression and constitutive activation of ALK protein [7], [8]. Subsequent studies have shown that about 20% of ALK+ ALCLs contain variant translocations in which the *ALK* gene is fused to other partner genes [7], [9]. ALK-fusion proteins interact with many adaptor proteins and activate several key signaling pathways involved in cell proliferation, transformation and survival, including STAT3, AKT/mTOR and ERK1/2 signaling pathways [8], [10], [11]. Furthermore it was shown that NPM-ALK exerts HuR-mediated posttranscriptional control on C/EBPβ gene expression that leads to increased C/EBPβ mRNA stability and translation in ALK+ ALCL [12].

In our previous study, we showed that the expression of C/EBPβ in ALK+ ALCL is controlled primarily by the STAT3 pathway, whereas its phosphorylation and activation is partially dependent on the MAPK pathway. Furthermore, we demonstrated a critical role of C/EBPβ in the proliferation and survival of ALK+ ALCL cells [6]. Because C/EBPβ seems to be central to ALK transformation, the aim of the current study was to identify downstream targets of C/EBPβ to gain insight in the pathogenesis of ALK+ ALCL. We now demonstrate using gene expression profiling (GEP) and chromatin immunoprecipitation (ChIP) analyses that C/EBPβ regulates important genes responsible for cell proliferation and survival in ALK+ ALCL. We explore in some detail, one of these genes, the RNA helicase DEAD box polypeptide 21 (DDX21), a member of the DExD/H box family of proteins that play important roles in RNA metabolism.

## Methods

### Ethics Statement

Ethics approval for the study (620/2011BO2) was obtained from the Ethics Committee at the Medical Faculty, University Tübingen. Written informed consent was obtained for the lymphoma specimens used in this study.

### Plasmids, Cell Culture and Patient Samples

Oligonucleotides containing the C/EBPβ-shRNA [6] and DDX21-shRNA (5′-TGATAAGACTGAAGAGATA-3′) sequences were cloned into pSuper (Oligoengine, Seattle, WA, USA) and pFUGW as previously described [6], [13]. pGIPZ lentiviral vectors containing a BCL2A1- and G0S2-shRNA were purchased from Open Biosystems (Open Biosystems Products/Thermo Fisher Scientific, Huntsville, AL, USA). The In-Fusion™ Advantage PCR Cloning Kit (Clontech Laboratories, Mountain View, CA, USA) was used to clone the template for the expression of the C/EBPβ isoforms LAP* and LAP into the vector pRRL.PPT.SF.i2GFPp [14] (for details see Methods in File S1). The ALK+ ALCL cell lines (SUDHL-1, KiJK and Karpas 299) were provided by Mark Raffeld (National Cancer Institute, NIH, Bethesda, MD, USA), and cultured as recently described [6]. SUDHL-1 and Karpas 299 were purchased from the American Type Culture Collection (ATCC) and KiJK was obtained from the author [15]. All three cell lines have been authenticated and are suitable for *in vitro* model system for ALCL [16]. The ALK- ALCL cell lines Mac-1 was provided by Eva Geißinger (University of Würzburg, Germany), and Mac-2A by Olaf Merkel (Deutsches Krebsforschungszentrum (DKFZ), Heidelberg, Germany). The mantle cell lymphoma (MCL) cell line Granta 519 was obtained from the cell bank of the mantle cell lymphoma research initiative and consortium after authentication and now is available from ATCC. Eight ALK- and ten ALK+ ALCL primary samples were collected from the files of the Institute of Pathology (University Hospital Tübingen, Germany). All cases were comprehensively immunophenotyped, as part of the diagnostic work-up, and were classified following the recommendations of the World Health Organization classification for tumors of haematopoietic and lymphoid tissues [9].

### Virus Production and Viral Infections

Production of virus containing pFUGW was performed as recently described [17]–[19]. Production of virus containing the pGIPZ vectors was performed transfecting 3××10^6^ cells in 10 cm plates using 30 µl TransIT 2020, 2 µg pHCMV-G, 6 µg pCMVdeltaR8.9 and 8 µg of the respective pGIPZ derivate according to the manufacturers manual (Mirus Bio, Madison, MI, USA). Production of virus containing the pRRL.PPT.SF.i2GFPp vectors was also performed (see Methods in File S1). Infection of lymphoma cells was performed as recently described with modified centrifugation conditions (800 g, 32°C, 90 min) [17]. To achieve efficient knockdown of *DDX21*, infection was repeated after 24 h (MOI of 90). Transduction efficiency was determined as previously specified [6], [17].

### Cell Proliferation and Viability Assay (MTS Assay) and Analysis of Apoptosis

Cell viability and growth retardation was determined by the MTS cell proliferation assay (AQueous CellTiter 96, Promega, Mannheim, Germany) [6]. Cell cycle analysis by flow cytometry was performed according to Nuesse et al. (FACSCalibur, BD, Franklin Lakes, NJ, USA) [20]. Analysis of apoptosis was done by annexin V (AnnexinV-APC, Invitrogen) and propidium iodide (PI) (Sigma-Aldrich, St. Louis, MO, USA) stainings according to standard protocols, followed by flow cytometry.

### Western Blot Analysis

Cells were lysed, as previously described [5], [6], [17]. Some protein fractions were enriched using NE-PER Nuclear and Cytoplasmic Extraction Reagents (Thermo Fisher Scientific, Waltham, MA, USA). For immunoblotting the following antibodies were used: C/EBPβ C-19 (1∶200, Santa Cruz Biotechnology, Santa Cruz, CA, USA), DDX21 (1∶1000, ProteinTech Group, Chicago, IL, USA), G0S2 (1∶500, Sigma-Aldrich), BCL2A1 (1∶250), TRIB1 (1∶1500) (abcam, Cambridge, UK), caspase 3 and cleaved caspase 3 (1∶1000, Cell Signaling, Frankfurt, Germany). The α-tubulin antibody (1∶5000, Sigma-Aldrich) was used as loading control. All experiments were repeated several times and representative data are shown.

### RNA Isolation and GEP

Total RNA was isolated using the RNeasy Mini kit (Qiagen, Hilden, Germany) including DNase treatment in cell lines and phenol-chloroform extraction for formalin-fixed paraffin embedded tissues in primary cases [21]. For GEP RNA quality was examined using the Agilent 2100 Bioanalyzer and only high quality RNA (RIN>8) was used for subsequent analyses.

Three biological replicates (transduction with control shRNA or shRNA directed against C/EBPβ) were used for the analysis. Total RNA (1 µg) was isolated 72 hours after transduction and amplified using a single round amplification kit (Affymetrix, Santa Clara, CA, USA). 10 µg of amplified aRNA were hybridized on Affymetrix U133 plus 2.0 arrays containing about 54.000 probe sets. Staining and scanning was done according to the Affymetrix standard protocol. The Bioconductor software package implemented in CARMAweb [22] was employed to calculate probe set summaries (RMA) and for normalization using the quantile algorithm. For statistical analysis the moderated limma test was applied in combination with the Benjamini-Hochberg correction for multiple testing. Probe Sets with a False Discovery Rate (FDR)<10% were considered as significant. Heatmaps and dendrograms were generated with tools provided by CARMAweb.

Gene Ontology (GO) terms [23] and pathway analysis were done with the Genomatix Pathway System (Genomatix, Munich, Germany) and Ingenuity IPA software was used to create one of the figures. Microarray data has been submitted to GEO (GSE23368): http://www.ncbi.nlm.nih.gov/geo/query/acc.cgi?token=hxoppwqssooewvo&acc=GSE23368.

### Real-time Quantitative RT-PCR (RT-qPCR)

*C/EBPβ* mRNA levels were quantified using *TBP* as housekeeping gene as previously specified [6], [24]. For all other genes, primers and probes were combined applying the Universal ProbeLibrary System (Roche Applied Science, Penzberg, Germany) (Table S1 in File S1). 500 ng RNA were transcribed into cDNA using Superscript II reverse transcriptase (Invitrogen, Carlsbad, CA, USA) and a mix of Oligo(dT) primer (Promega, Madison, WI, USA) and random hexamers (Roche Applied Science, Penzberg, Germany) in a final volume of 50 µl according to the manufacturer’s instructions. RT-qPCRs were carried out in duplicates with the LightCycler 480 Probes Master using a LightCycler 480 System for detection (Roche Applied Science). Data were analyzed using the 2^−ΔΔCp^ method [5].

### Chromatin Immunoprecipitation (ChIP) Assay and Luciferase Reporter Assay

SUDHL-1 cells were fixed, nuclei were isolated and chromatin was sheared by sonication using the ChIP-IT Express kit according to the manufacturer’s instructions (Active Motif, Carlsbad, CA, USA). Sheared chromatin was immunoprecipitated using C/EBPβ antibody (3 µg, Santa Cruz Biotechnology) and IgG (3 µg, Sigma-Aldrich). Isolated DNA was analyzed by qPCR (LightCycler 480 (Roche Applied Science)), and amplification of 50 sequences (primers Table S2 in File S1) of 11 genes (*BCL2A1, TRIB1, G0S2, S100A9, DDIT4, TM4SF1, CDKN2A, PTPRC, DDX21, JUN, CCL20*) with potential C/EBPβ binding sites (Bibliosphere, Genomatix, TFSEARCH (http://www.cbrc.jp/research/db/TFSEARCH.html)) was performed using the QuantiTect Sybr Green PCR Kit (Qiagen) according to the manufacturer's protocol. Some qPCRs covered more than one regulatory site. To normalize for variations in the amount of starting DNA, a ΔCp value was calculated (ΔCp(input)-ΔCp(ChIP)). DNA quantities were expressed as percentages of corresponding input (2^−ΔΔCp^x100). Mean values of three biological replicates were calculated. Statistical analysis was carried out using *t*-test. To get additional evidence for C/EBPβ directly targeting the regulatory site of the intron region of *DDX21,* luciferase reporter assays were performed (see Methods in File S1).

### Immunohistochemistry

All primary cases were previously studied in paraffin sections with a panel of antibodies to assess tumor cell phenotype. Additionally, the expression of C/EBPβ (C-19; Santa Cruz Biotechnology, 1∶200), ALK (1∶400), CD30 (1∶50) (Dako, Glostrup, Denmark), BCL2A1 (abcam, Cambridge, UK, 1∶25) and DDX21 (ProteinTech Group, 1∶500) was investigated in ten ALK+ and eight ALK- ALCL cases. Immunohistochemical analysis was performed on the Ventana Benchmark automated staining system using Ventana reagents. Primary antibody detection was performed using the iView™ diaminobenzidine detection kit (Ventana Medical Systems, Tucson, AZ, USA). Photomicrographic images were acquired with an Axioskop 2 *plus* Zeiss microscope equipped with a Jenoptik (Laser Optik System, Jena, Germany) ProgRes C10 *plus* camera and software. Objectives Plan-Neofluar used were: 1.25/0,035, 2.5x/0.075, 10x/0.30, 20x/0.50, and 40x/0.75. Final image preparation was performed with Adobe Photoshop CS2.

### Metabolic Labeling of Nascent Pre-rRNA with 32P

SUDHL-1 cells were metabolically labeled with ^32^P after transduction with DDX21-shRNA. 1.5×10^6^ cells were cultured with phosphate-free RPMI 1640 (MP Biomedicals, Singapore) for 3 h. Medium was replaced with phosphate-free medium containing 40 µCi/ml [^32^P]Orthophosphate (PerkinElmer, Waltham, MA, USA) and cells were incubated for an additional 1.5 h. The chase experiment was done by cultivating the labeled cells with nonradioactive standard RPMI 1640 supplemented with 10% FCS at 0, 2, 3.5 and 5 h. Total RNA was isolated using the RNeasy Mini kit and 250 ng of total RNA was resolved on a formaldehyde-containing 1% agarose gel. The gel was dried and radioactive bands were detected by autoradiography.

## Results

### Efficient Transfer of C/EBPβ-shRNA into ALCL Cell Lines Using a Lentiviral Vector

**Figure 1:**
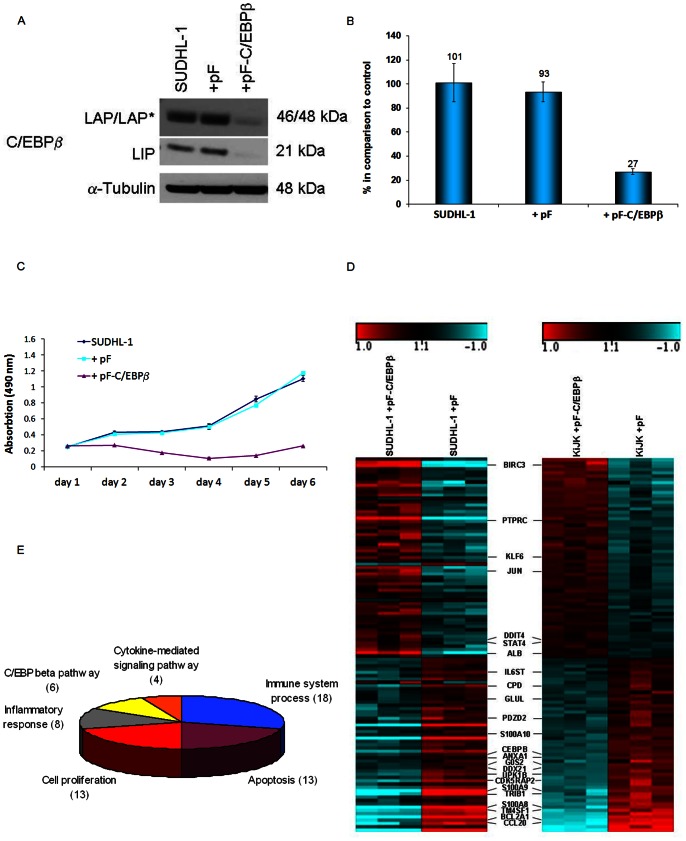
Lentiviral transduction of C/EBPβ shRNA in SUDHL-1 cell line and gene expression profiling. (**A**) Western Blot analysis of the different C/EBPβ isoforms (liver-enriched activation protein (LAP*, LAP), liver-enriched inhibitory protein (LIP) in the transduced SUDHL-1 cells three days after infection demonstrates successful knockdown. Each lane contains 40 µg protein extract. α-Tubulin was used as loading control. (**B**) RT-qPCR analysis of *C/EBPβ* mRNA in the transduced SUDHL-1 cells three days after infection. Values were normalized to *TBP* and data were analyzed according to the 2^−ΔΔCp^ method. Results are depicted as mRNA amount relative to untreated SUDHL-1 cells. Error bars indicate SD (n = 4). (**C**) Proliferation curves of the controls and C/EBPβ-shRNA infected SUDHL-1 cells are depicted up to 6 days after infection. Error bars indicate SD (n = 3). (**D**) *C/EBPβ* signature of SUDHL-1 and KiJK cells derived by C/EBPβ-shRNA. Heatmaps show the expression pattern of the 114 genes which were significantly (FDR<10%) regulated after *C/EBPβ* knockdown in both cell lines, SUDHL-1 and KiJK, depicting transduced cells with C/EBPβ-shRNA and controls in triplicates. The 23 candidate genes whose regulation was validated by RT-qPCR are designated. The coloured log2 scale bar represents relative gene expression changes. (**E**) GO term enrichment analysis was used to assess the biological functions of the 114 differentially expressed genes [23]. Selected terms (p<0.01) and the number of genes associated with them are shown. SUDHL-1 = uninfected cells, pF = empty virus, pF-C/EBPβ = virus containing the C/EBPβ shRNA sequence.

Two ALK+ ALCL cell lines with strong C/EBPβ expression – SUDHL-1 and KiJK – were transduced with C/EBPβ-shRNA. Flow cytometric analysis showed that C/EBPβ-shRNA was effectively transduced into the cell lines with a high infection rate of almost 100%. Three days after transduction, C/EBPβ was successfully downregulated, as demonstrated by Western blot and RT-qPCR (Figure 1A–B and Figure S1A–B). *C/EBPβ* mRNA was reduced by 73% compared to untreated control cells. To further evaluate the effect of C/EBPβ downregulation, growth curves were generated from treated cells up to six days after infection. A comparison between control and C/EBPβ-shRNA-infected cells on day six of infection revealed growth retardation of 81.3% in SUDHL-1 (Figure 1C), and of 72.4% in KiJK (Figure S1C).

### Identification of Differentially Expressed Genes After C/EBPβ Downregulation

In order to identify the downstream targets of C/EBPβ in ALK+ ALCL that might be responsible for survival and proliferation, GEP was performed after C/EBPβ silencing in the two ALK+ ALCL cell lines SUDHL-1 and KiJK, and compared with mock-treated cells. Statistical analysis of the gene expression data resulted in 595 genes (corresponding to 909 probe sets) that were significantly (FDR<10%) regulated in SUDHL-1 after C/EBPβ knockdown (314 upregulated, 278 downregulated, 3 ambigous). In KiJK cells, 392 significantly regulated genes (262 upregulated, 130 downregulated; corresponding to 582 probe sets) were identified. Among the significantly regulated genes 129 were regulated more than 2-fold in SUDHL-1 and 61 in KiJK. To define a consistent signature of C/EBPβ-dependent transcripts, we focused on genes that were significantly regulated after *C/EBPβ* silencing in both cell lines. The majority of these 114 genes (corresponding to 166 probe sets, Table S3 in File S1) showed similar regulation (up or down) in SUDHL-1 and KiJK (Figure 1D). To address the biological functions of the 114 differentially expressed genes, we analyzed their associated GO terms to obtain significantly (p<0.01) enriched terms (Table S4 in File S1) [23]. This analysis revealed the overrepresentation of terms related to immune response, apoptosis and cell proliferation (Figure 1E).

**Figure 2:**
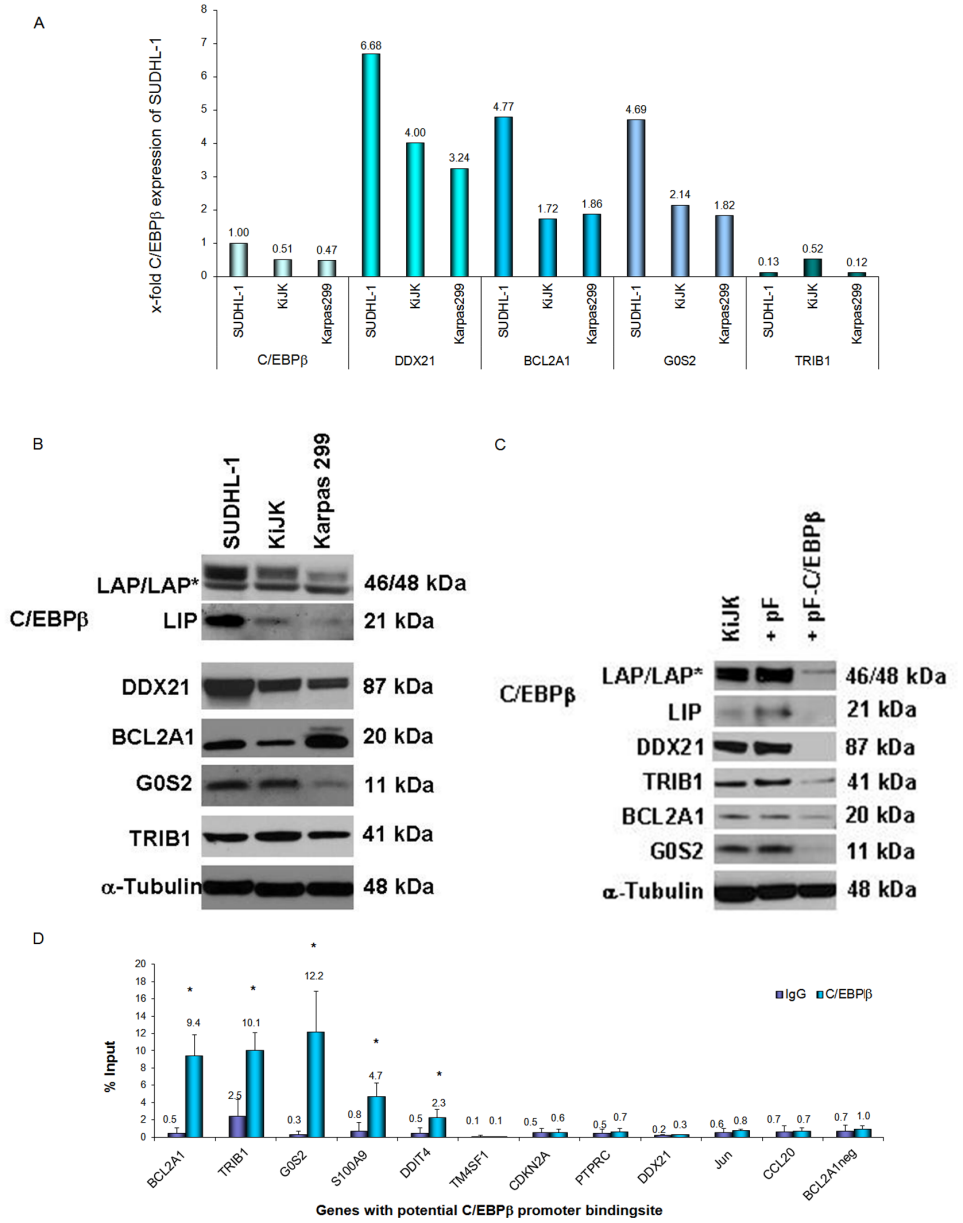
Expression and ChIP analysis of selected candidate genes in ALK+ ALCL cell lines. (**A**) RT-qPCR analysis of *C/EBPβ*, *DDX21*, *BCL2A1*, *G0S2* and *TRIB1* in SUDHL-1, KiJK and Karpas 299 cell lines. Values were normalized to *TBP* and data were analyzed according to the 2^−ΔΔCp^ method. Results are depicted as mRNA amount relative to the *C/EBPβ* mRNA expression level in SUDHL-1 cells. (**B**) C/EBPβ, DDX21, BCL2A1, G0S2 and TRIB1 expression in SUDHL-1, KiJK and Karpas 299 detected by Western Blot. Each lane contains 40 µg protein extract. (**C**) Western Blot analysis of C/EBPβ, DDX21 and BCL2A1 in KiJK cell transduced with C/EBPβ shRNA four days after infection. 20 µg cytoplasmic extracts were loaded to detect G0S2 and BCL2A1 and 8 µg nuclear protein extracts were loaded to detect C/EBPβ, DDX21 and TRIB1. α-Tubulin was used as loading control. (**D**) Representative results of qPCR analyses to quantify the relative amounts of DNA obtained by ChIP for the potential regulatory sites of *BCL2A1*, *TRIB1*, *G0S2*, *S100A9*, *DDIT4*, *TM4SF1*, *CDKN2A*, *PTPRC*, *DDX21*, *JUN*, *CCL20*. DNA quantities (expressed as percentages of input) were compared for specific IPs versus IgG IP samples. One representative qPCR result of the different sites of each gene is shown. Error bars indicate SD (n = 3), p<0.05.

To identify the most promising candidates of C/EBPβ-mediated oncogenesis, 26 genes that were strongly regulated (14 genes) and/or had potential C/EBPβ promoter binding sites (17 genes) (Bibliosphere, TFSEARCH (http://www.cbrc.jp/research/db/TFSEARCH.html)) were selected for further analysis. Validation of the candidate genes by RT-qPCR confirmed the regulation of 23 of the 26 genes in both cell lines (Table 1). Some of these genes showed remarkable pathway connections, as revealed by the “Ingenuity pathway” program. The three best candidate genes - *G0S2*, *DDX21 and BCL2A1*– showed very high mRNA expression levels (Figure 2A), and followed the same pattern as *C/EBPβ* expression in the three analyzed ALK+ ALCL cell lines. SUDHL1 showed the highest mRNA expression levels of all three genes, whereas Karpas 299 showed the lowest levels of expression. Western blot analysis corroborated the expression of BCL2A1, DDX21 and G0S2 in all three analyzed ALK+ ALCL cell lines. Additionally, *TRIB1* was expressed in all three cell lines but at lower levels (Figure 2B). To further validate the C/EBPβ dependency of these genes, Western blot analysis was performed after C/EBPβ silencing. Coordinate with the reduction of C/EBPβ protein, there was a clear decrease in the protein levels of BCL2A1, DDX21, G0S2 and TRIB1 (Figure 2C) with the strongest correlation observed for DDX21. This suggested that these four genes were regulated by C/EBPβ.

**Table 1:** Genes differentially expressed after *C/EBPβ* knockdown.

Genes	SUDHL-1 ratio array	SUDHL-1 ratio qPCR	KiJK ratio array	KiJK ratio qPCR
BCL2A1	0.39	0.28	0.29	0.26
DDX21	0.66	0.49	0.52	0.49
G0S2	0.63	0.71	0.5	0.55
TRIB1	0.23	0.13	0.42	0.44
CCL20	0.3	0.55	0.27	0.4
TM4SF1	0.44	0.25	0.35	0.25
UPK1B	0.42	0.41	0.45	0.21
ANXA1	0.75	0.69	0.51	0.57
S100A10	0.67	0.52	0.57	0.38
GLUL	0.69	0.59	0.61	0.79
S100A9	0.19	0	0.42	0.38
PDZD2	0.48	0.57	0.59	0.07
CPD	0.44	0.41	0.57	0.67
S100A8	0.09	0.01	0.32	0.25
IL6ST	0.59	0.55	0.65	0.79
CDK5RAP2	0.37	0.26	0.48	0.55
*CDKN2A*	*0.56*	*0.82*	*0.42*	*1*
*PLA2G4A*	*0.4*	*0.38*	*0.44*	*1.18*
*NCOA7*	*0.37*	*1.11*	*0.53*	*0.79*
JUN	1.83	3.14	1.58	1.68
BIRC3	3.88	3.33	2.13	3.03
DDIT4	1.59	2.4	1.41	1.73
STAT4	1.58	1.47	1.4	1.27
PTPRC	2.43	2.46	1.65	1.72
KLF6	2.14	1.88	1.7	1.7
ALB	3.61	2.17	1.38	1.16

Array and qPCR expression level ratios (shRNA vs. control) of cells infected with empty virus and C/EBPβ-shRNA.

The regulation by C/EBPβ knockdown of three genes that could not be confirmed by qPCR is in italics.

### Direct Gene Targets of C/EBPβ

**Figure 3:**
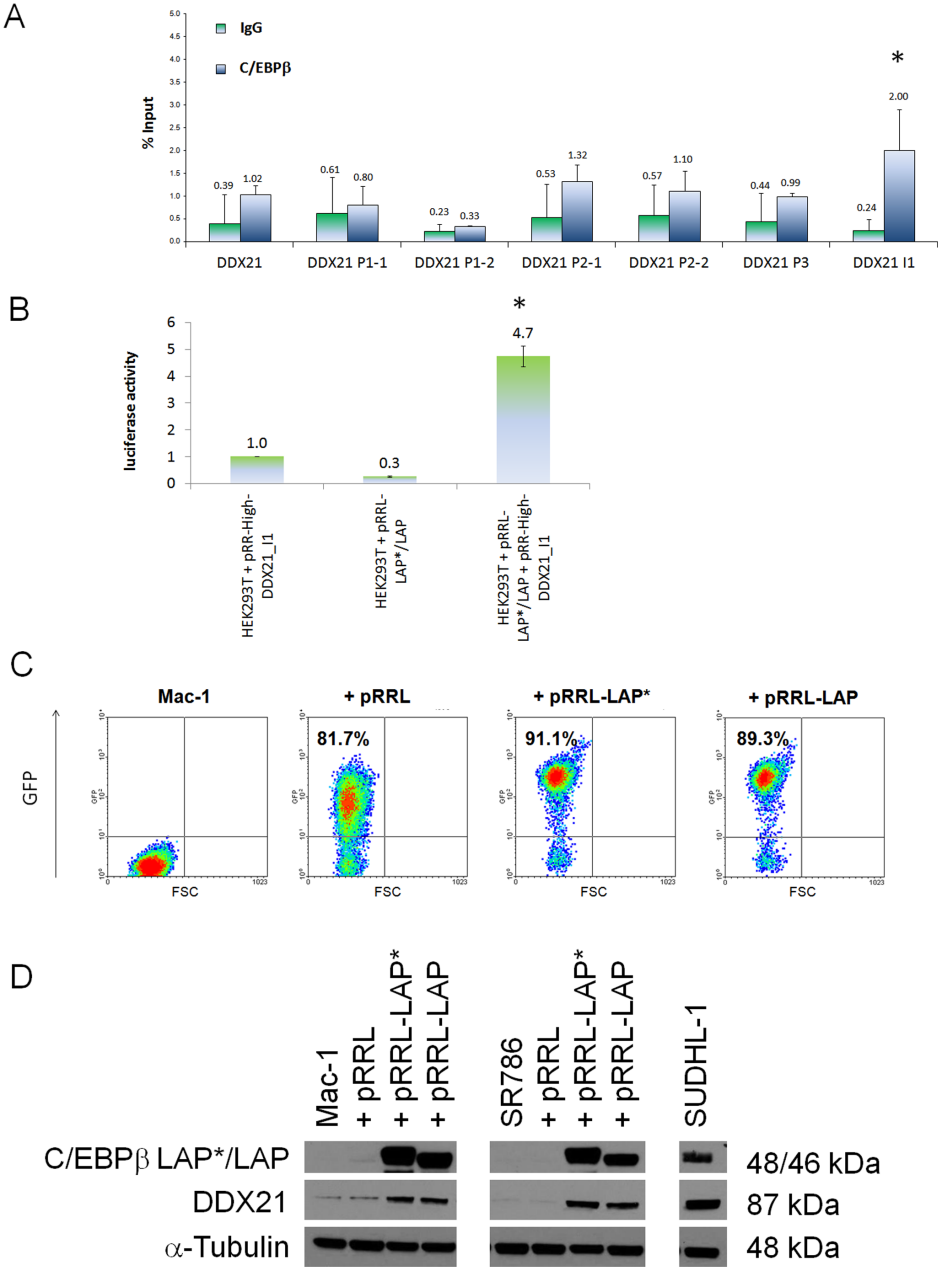
ChIP analysis of DDX21 gene, luciferase reporter assay and overexpression of C/EBPβ. (**A**) qPCR analysis to quantify the relative amounts of DNA obtained by ChIP for the potential regulatory sites of *DDX21*. DDX21_I1 depicts the regulatory site in the first intron of the *DDX21* gene. DNA quantities (expressed as percentages of input) were compared for specific IPs versus IgG IP samples. One representative qPCR result of the different sites is shown. Error bars indicate SD (n = 3), p<0.05. (**B**) Luciferase reporter assays after transfection of HEK293T cells with the reporter plasmid containing the regulatory site of the intron region of *DDX21* and expression vector to overexpress the C/EBPβ isoforms LAP* and LAP. Bars represent relative luciferase activity for the respective vectors. Error bars indicate SD (n = 3), p<0.05. (**C**) Flow cytometric analysis of transduced Mac-1 cells and untreated controls three days after lentiviral infection. The percentage of GFP-positive cells represents the percentage of infected cells. (**D**) Western Blot analysis of the C/EBPβ isoforms LAP* and LAP and of DDX21 in the transduced Mac-1 and SR786 cells three days after infection. Each lane of the Western Blot contains 20 µg protein extract. Tubulin was used as loading control. Mac-1/SR786 = uninfected cells, pRRL = empty virus, pRRL-LAP* = virus containing the C/EBPβ isoform LAP* sequence, pRRL-LAP = virus containing the C/EBPβ isoform LAP sequence.

We next investigated 50 potentially transcriptional regulatory locations (Table S2 in File S1) of eleven genes, which had been selected using the web tool TFSEARCH to predict proximal promoter elements and Bibliosphere to predict regulatory elements that can be distributed further over the respective gene locus, to identify C/EBPβ binding sites by qPCR after ChIP analysis in SUDHL-1 cells. C/EBPβ binding was detected on at least one site of the regulatory regions of *BCL2A1*, *TRIB1*, *G0S2*, *S100A9* and *DDIT4* consistent with direct regulation by C/EBPβ. Representative qPCR results of the different regulatory sites of each gene are shown in Figure 2D. C/EBPβ binding was detected in more than one location in several genes. Positive signals were observed in three of four different regulatory regions of the *BCL2A1* gene, two of three locations within the *G0S2* gene and two of seven locations within the *S100A9* gene, whereas C/EBPβ binding was detected in a single regulatory region of *TRIB1* and *DDIT4*. Surprisingly, no signals were detected analyzing the predicted *DDX21* sites, although *DDX21* was a promising candidate gene for direct regulation by C/EBPβ because of its high mRNA and protein expression and strong correlation with C/EBPβ expression in ALK+ ALCL cells. However, all six potential binding sites in the proximal region −1585 to −130 5′ of the transcriptional start site of the *DDX21* gene investigated were negative (Figure 3A). Considering that regulatory elements could have escaped the Bibliosphere screening, the TFSEARCH analysis was extended to intron regions of the *DDX21* gene. An additional regulatory site in the first intron of the gene was found (Table S2 in File S1) that showed a significant positive signal by ChIP analysis (Figure 3A), providing strong evidence for the direct transcriptional regulation of *DDX21* by C/EBPβ. To further demonstrate that *DDX21* is a direct target of C/EBPβ, luciferase reporter assay was performed analyzing the regulatory site located in the first intron of *DDX21*. Overexpression of C/EBPβ (transient expression of the complete coding sequence produces mainly isoform LAP* and LAP) in HEK293T cells led to a significant increase in luciferase activity in comparison to the transfection of each of the plasmids separately (Figure 3B). To further confirm the C/EBPβ-dependent expression of DDX21, the isoforms LAP* and LAP, which have an activating transcriptional function, were overexpressed in the ALK- ALCL cell line Mac-1 and the ALK+ ALCL cell line SR786, which does not express C/EBPβ. Lentiviral vectors containing the coding sequence of LAP* and LAP were efficiently transduced in the cells, as demonstrated by expression of the GFP reporter gene (Figure 3C). In both cell lines a strong upregulation of the isoforms LAP* and LAP was detected three days after infection together with clear upregulation of DDX21 (Figure 3D). Taken together, these data confirm that D*DX21* is a direct target of C/EBPβ.

qPCRs of the potential regulatory sites of the remaining five genes investigated (*TM4SF1, CDKN2A, PTPRC, JUN, CCL20*) showed no signals, indicating the absence of C/EBPβ binding. Consequently, these genes seem to be regulated by C/EBPβ by alternative mechanism(s) other than direct transcriptional activation.

### BCL2A1, G0S2 and DDX21 Contribute to the Survival and Proliferation of ALK+ ALCL Cells

**Figure 4:**
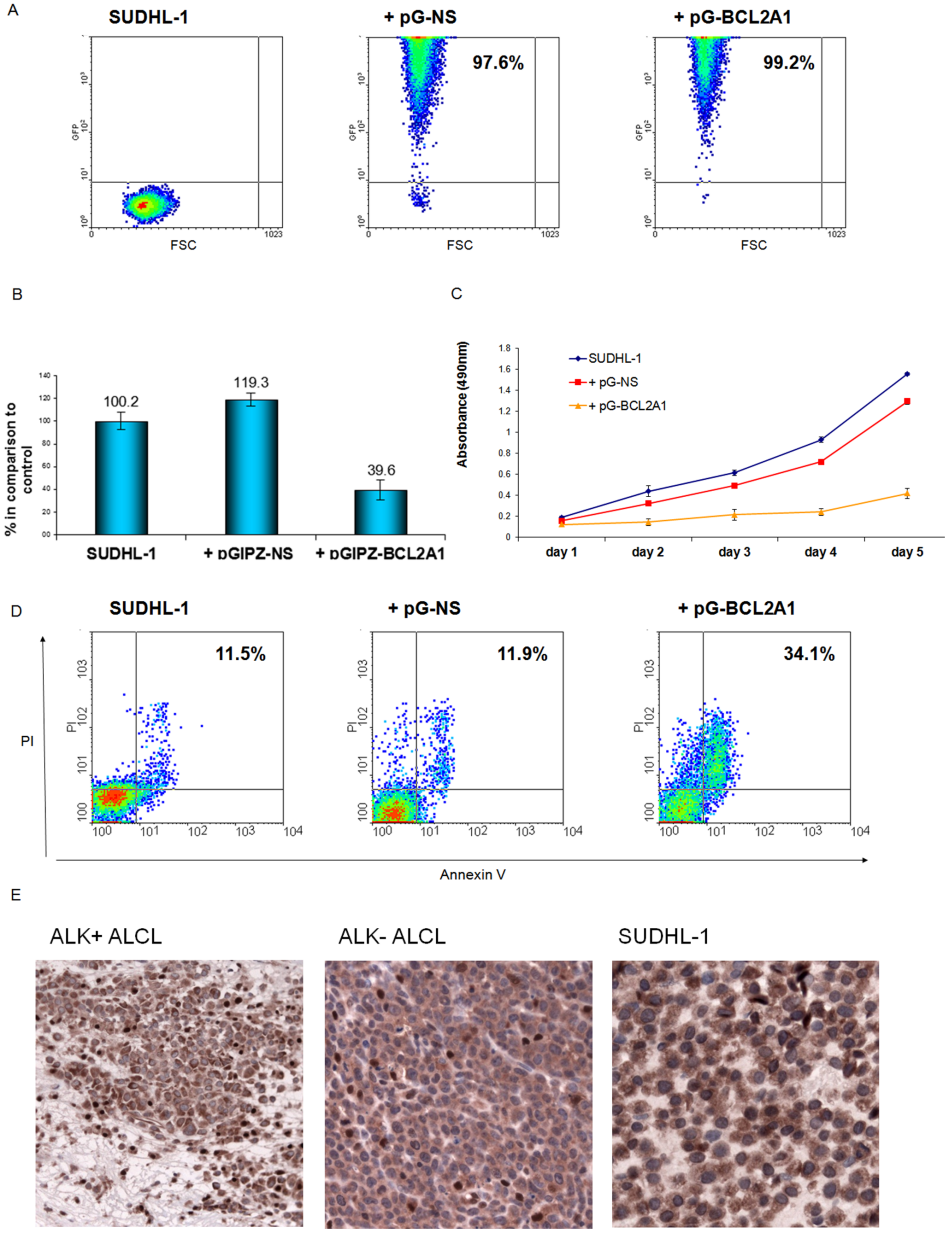
Lentiviral transduction of BCL2A1-shRNA in SUDHL-1 cells and its influence on cell proliferation and apoptosis. (**A**) Flow cytometric analysis of transduced SUDHL-1 cells and untreated controls three days after infection. The percentage of GFP-positive cells represents the percentage of infected cells. (**B**) RT-qPCR analysis of *BCL2A1* mRNA in the transduced SUDHL-1 cells three days after infection. Values were normalized to *TBP* and data were analyzed according to the 2^−ΔΔCp^ method. Results are depicted as mRNA amount relative to untreated SUDHL-1 cells. Error bars indicate SD (n = 3). (**C**) Proliferation curves of the controls and BCL2A1-shRNA infected SUDHL-1 cells up to 5 days after infection. Error bars indicate SD (n = 3). (**D**) Annexin V/propidium iodide staining of the controls and the BCL2A1-shRNA transduced SUDHL-1 cells three days after infection. SUDHL-1 = uninfected cells, pG-NS = pGIPZ vector with non-silencing shRNA, pG-BCL2A1 = virus containing the pGIPZ vector and the BCL2A1-shRNA sequence, PI = propidium iodide. (**E**) BCL2A1 immunostaining in ALCL. Representative ALK+ (left) and ALK- (middle) ALCL cases showing both a similar cytoplasmic BCL2A1 positivity in the tumor cells comparable to the expression in SUDHL-1 cells. (Immunoperoxidase, 400x).

To evaluate the effect of BCL2A1 in ALK+ ALCL cells, *BCL2A1* was silenced with a specific BCL2A1-shRNA construct in SUDHL-1 cells (Figure 4A). RT-qPCR confirmed the *BCL2A1* mRNA downregulation of approximately 60% compared to control cells (Figure 4B). Growth curves were generated from treated cells with 99% infection rate up to 6 days after infection. Downregulation of *BCL2A1* mRNA led to a strong growth retardation of almost 60% after two days of infection when compared to control cells (Figure 4C). To see whether the downregulation of *BCL2A1* induces apoptosis, flow cytometric analysis with annexin V/propidium iodide was performed three days after infection. BCL2A1-shRNA-infected cells revealed increased cell death (34.1%) when compared to the control cells (11.5% and 11.9% respectively) (Figure 4D). These findings demonstrate the important role of BCL2A1 in the survival and sustained growth of ALK+ ALCL cells. To investigate whether BCL2A1 was also expressed in primary cases, we analyzed 10 ALK+ and 8 ALK- ALCL cases by immunohistochemistry (Figure 4E). All cases analyzed were similarly positive for BCL2A1, suggesting that the overexpression of BCL2A1 is important for promoting survival in both tumor types.

**Figure 5:**
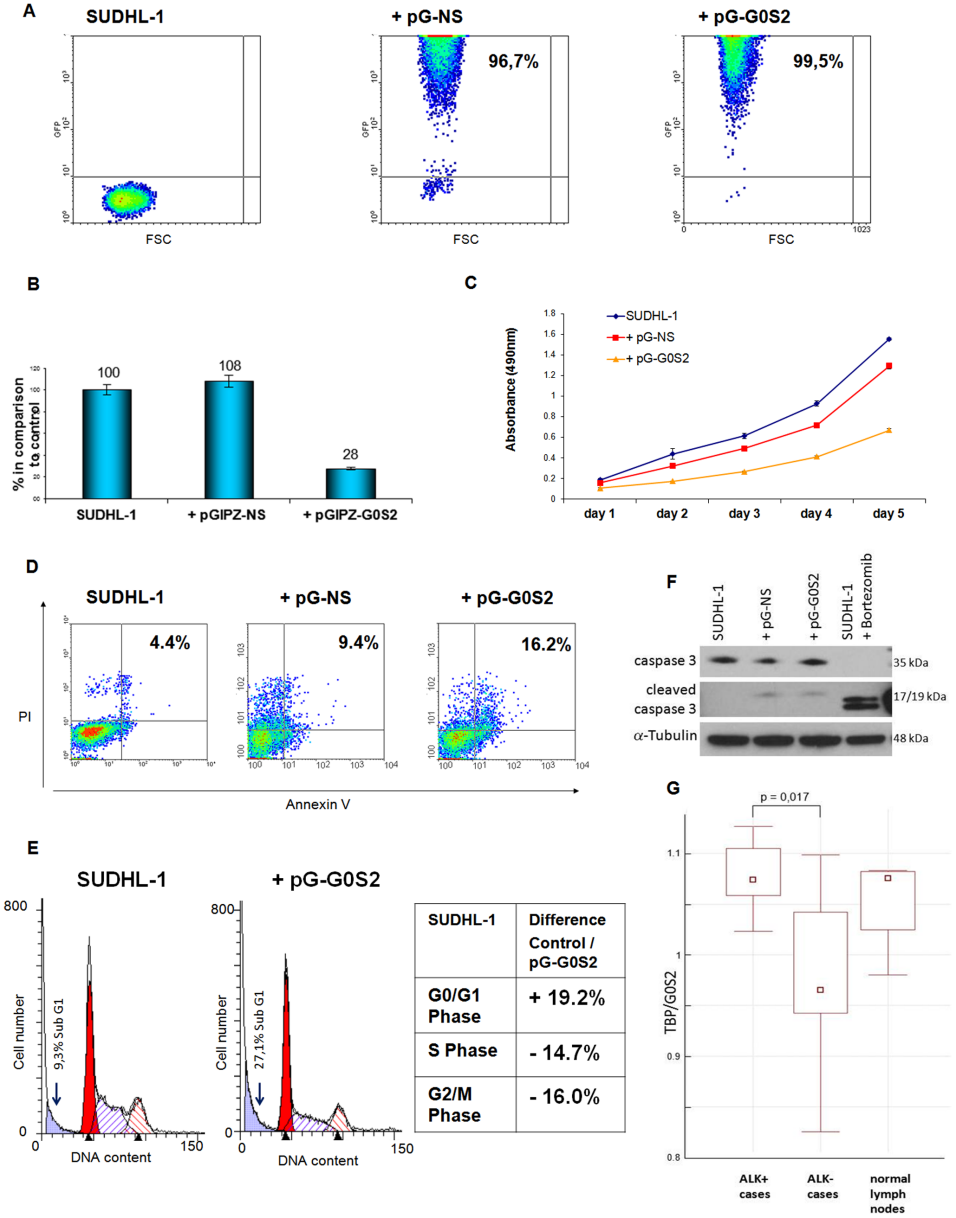
Influence of G0S2 knockdown on cell proliferation, apoptosis and cell cycle. Validation in primary cases. (**A**) Flow cytometric analysis of transduced SUDHL-1 cells and untreated controls four days after infection. The percentage of GFP-positive cells represents the percentage of infected cells. (**B**) RT-qPCR analysis of *G0S2* mRNA in the transduced SUDHL-1 cells four days after infection. Values were normalized to *TBP* and data were analyzed according to the 2^-ΔΔCp^ method. Results are depicted as mRNA amount relative to untreated SUDHL-1 cells. Error bars indicate SD (n = 3). (**C**) Proliferation curves of the controls and G0S2-shRNA infected SUDHL-1 cells up to 5 days after infection. Error bars indicate SD (n = 3). (**D**) Annexin V/propidium iodide staining of the controls and the G0S2-shRNA transduced SUDHL-1 cells four days after infection. (**E**) Cell cycle distribution of the control and G0S2-shRNA infected SUDHL-1 cells four days after infection. The differences between the uninfected control and the G0S2-shRNA-infected cells are given in percentage in the table. SUDHL-1 = uninfected cells, pG-NS = pGIPZ vector with non-silencing shRNA, pG-G0S2 = virus containing the pGIPZ vector and the G0S2-shRNA sequence, PI = propidium iodide. (**F**) Western Blot analysis of caspase 3 and cleaved caspase 3 in G0S2-shRNA transduced SUDHL-1 cells four days after infection. Each lane contains 30 µg cytoplasmic extract. 15 µg protein extract of Bortezomib-treated SUDHL-1 cells were used as control. α-Tubulin was used as loading control. (**G**) Box plot of G0S2 expression levels obtained by RT-qPCR analysis in 6 ALK+, 8 ALK- and 4 normal lymph node samples.

Since *G0S2* has been implicated in cell cycle control, and was one of the genes with the highest mRNA expression, its role in the survival and proliferation of ALK+ ALCL cells was investigated. *G0S2* was silenced in SUDHL-1 cells with a specific G0S2-shRNA construct (Figure 5A). The knockdown of *G0S2* resulted in a 72% reduction of mRNA as demonstrated by RT-qPCR (Figure 5B). Downregulation of *G0S2* in SUDHL1 cells resulted in growth retardation of 23% after 5 days of infection when compared to the untreated control cells (Figure 5C). Only a slight increase in apoptosis was detected after GOS2 knockdown, as demonstrated by annexin V/propidium iodide assay (16.2% vs. 4.4%) (Figure 5D). Cell cycle analysis demonstrated a decrease in S and G2/M phase of 14.7% and 16.0%, respectively, and an increase in G0/G1 of 19.2% after four days of infection (Figure 5E). The subG1 fraction showed 27,1% of dead cells in the GOS2 downregulated cells compared to 9,3% in the untreated control cells. These results reflect the mild apoptosis found with the annexinV/propidum iodide assay. Western blot analysis for caspase 3 showed a mild increase in cleaved caspase 3 after GOS2 downregulation (Figure 5F). Taken together, these results show a role of G0S2 in sustaining proliferation of ALK+ ALCL cells and only a minor role in inducing apoptosis. The expression of G0S2 protein was investigated in primary cases; however, the available antibodies gave a non-specific reaction in paraffin-embedded tissue. Therefore, *G0S2* mRNA levels were analyzed in six ALK+ vs. eight ALK- primary cases (Figure 5G). ALK+ ALCL cases showed a significantly higher *G0S2* mRNA expression than ALK- cases (p = 0.017).

**Figure 6:**
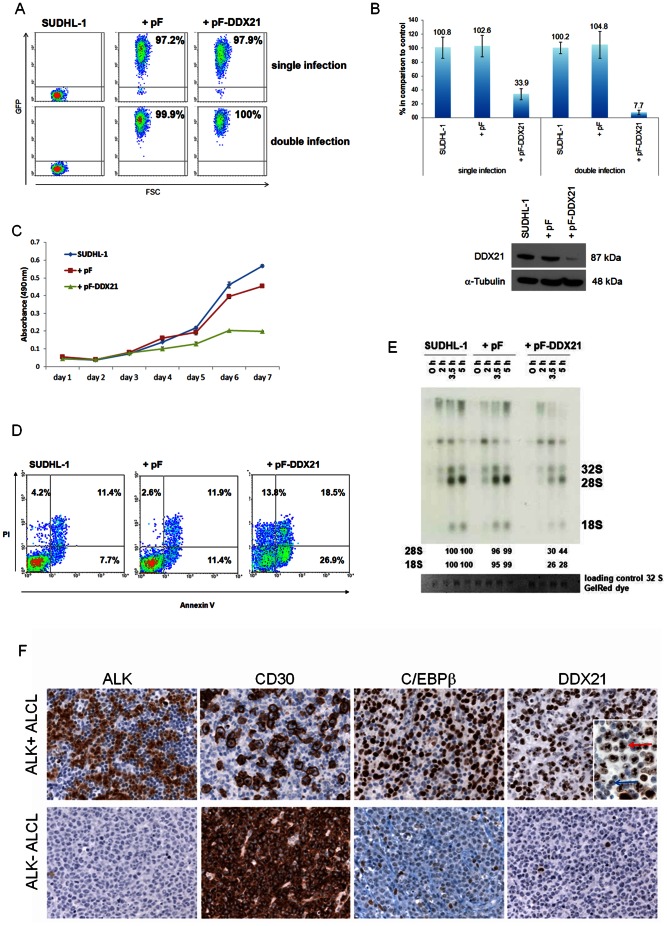
Influence of DDX21 knockdown on proliferation, apoptosis and pre-rRNA processing. DDX21 expression in primary cases. (**A**) Flow cytometric analysis of transduced SUDHL-1 cells and untreated controls three days after infection. The percentage of GFP-positive cells represents the percentage of infected cells. (**B**) Analysis of DDX21 mRNA and protein downregulation, investigated by RT-qPCR analysis of *DDX21* mRNA in the transduced SUDHL-1 cells three and four days after infection (upper part) and Western Blot analysis of C/EBPβ in the transduced SUDHL-1 cells four days after infection (lower part). RT-qPCR values were normalized to *TBP* and data were analyzed according to the 2^−ΔΔCp^ method. Results are depicted as mRNA amount relative to untreated SUDHL-1 cells. Error bars indicate SD (n = 3). Each lane of the Western Blot contains 30 µg protein extract. Tubulin was used as loading control. (**C**) Proliferation curves of the controls and DDX21-shRNA double-infected SUDHL-1 cells are depicted up to 7 days after infection. Error bars indicate SD. SUDHL-1 = uninfected cells, pF = empty virus, pF-DDX21 = virus containing the DDX21 shRNA sequence. (**D**) Annexin V/propidium iodide staining of the controls and the DDX21-shRNA transduced SUDHL-1 cells four days after infection. PI = propidium iodide. (**E**) Autoradiogram of rRNA pulse-labelled with [^32^P] orthophosphate and chased for the indicated time. SUDHL-1 cells transduced with DDX21-shRNA, with the empty virus and untreated control cells were compared. The levels of 28S and 18S rRNA relative to those of the samples of the untreated control cells are shown at the bottom. SUDHL-1 = uninfected cells, pF = empty virus, pF-DDX21 = virus containing the DDX21-shRNA sequence. (**F**) ALK, CD30, C/EBPβ and DDX21 immunostaining in ALCL. The ALCL primary case (upper panel), shows nuclear and cytoplasmic ALK expression, strong nuclear positivity for C/EBPβ and strong nucleolar positivity for DDX21 in most tumor cells. Insert: Higher magnification demonstrating the strong DDX21 nucleolar positivity of the tumor cells (red arrow), in contrast to the small reactive lymphocytes with normal nucleolar expression (blue arrow). The ALK-negative case (lower panel) is negative for C/EBPβ, with reactive histiocytes serving as internal positive control. DDX21 shows only weak nucleolar positivity. Immunoperoxidase, 400x (insert, 630x).

As *DDX21* had been proved to be involved in pre-rRNA processing and participated in cell growth [25]–[28], the possible role of *DDX21* in cell proliferation and survival as well as in rRNA processing of ALK+ ALCL cells was further investigated. SUDHL-1 cells were transduced with a specific DDX21-shRNA construct (Figure 6A). Because of the very high DDX21 mRNA expression levels, a second infection was performed after 24 hours, which resulted in a further mRNA reduction of 92% compared to 66% after the first infection (Figure 6B). Efficient downregulation of DDX21 mRNA and protein showed growth retardation of 65.3% in SUDHL-1 cells after 7 days (Figure 6C), indicating an important role of DDX21 for promoting proliferation and survival in ALCL cells. Flow cytometric analysis with annexin V/PI was performed 4 days after infection and revealed increased apoptosis and cell death in DDX21-shRNA-infected cells (26.9% annexin V+/PI-, 18.5% annexin V+/PI+ and 18.8% annexin V−/PI+) when compared to the controls (7.7/11.4% annexin V+/PI-, 11.4/11.9% annexin V+/PI+ and 4.2/2.6% annexin V−/PI+) (Figure 6D). To investigate whether the impact of DDX21 knockdown in ALK+ ALCL cells was unique to these cells, we performed DDX21 knockdown in other cell lines (Mac-2A, ALK- ALCL and the MCL Granta 519). The efficient downregulation of DDX21 mRNA and protein in these two cell lines after the first infection produced also growth retardation; however, the effect was stronger in Mac-2A cells (94.3%) than in in Granta 519 cells (64.1%) (Figure S2). These results suggest that DDX21 is necessary to maintain proliferation in different lymphoma cells. To analyze the possible implication of DDX21 in rRNA biogenesis and cell metabolism of ALK+ ALCL, we investigated whether processing of pre-rRNA was interrupted by silencing *DDX21*. Autoradiography of RNA purified from ALK+ ALCL cells with strong DDX21 knockdown demonstrated a clear delay in processing of 32S-labeled pre-rRNA. Downregulation of DDX21 resulted in a 70 and 74% decrease in the levels of 28S and 18S rRNA, respectively after a 3.5 h chase compared with the control cells (Figure 6E).

To determine whether the high expression of DDX21 was also present in primary cases, ten ALK+ and eight ALK- ALCL cases were analyzed by immunohistochemistry. All ALK+ ALCL cases showed a strong nucleolar expression of DDX21 in the tumor cells (Figure 6F). In contrast, ALK- ALCL cases showed normal expression of DDX21 comparable to the reactive lymphoid cells. These results clearly demonstrated differential expression of DDX21 in ALK+ ALCL compared to ALK- ALCL primary cases, and confirmed the strong interdependence of DDX21 and C/EBPβ expression observed in the cell lines.

## Discussion

We recently reported that NPM-ALK induces C/EBPβ expression primarily through the STAT3 signalling pathway and that C/EBPβ plays a central role in ALK-mediated transformation in ALK+ ALCL [6], [29]. In this study, we aimed to identify the downstream targets of *C/EBPβ* that might contribute to ALK-mediated lymphoid transformation. To achieve this objective, GEP was performed after *C/EBPβ* silencing in ALK+ ALCL cell lines. GEP produced a reliable and reproducible expression signature of 114 genes, which were significantly regulated by C/EBPβ in two ALK+ ALCL cell lines with strong C/EBPβ expression – SUDHL-1 and KiJK. Overrepresentation of genes mainly involved in immune system processes, apoptosis and cell proliferation reflected the central role of C/EBPβ in the proliferation and survival of ALK+ ALCL cells. Using ChIP analysis and highly effective shRNA sequences, we investigated the pathogenic role of several C/EBPβ-regulated genes and assessed their individual contribution to the maintenance of the malignant phenotype in ALK+ ALCL. We demonstrated that three genes, *BCL2A1, G0S2*, and *DDX21* are necessary for sustaining survival and proliferation of ALK+ ALCL cells, and found several other potentially interesting genes.

GEP is a useful tool not only in defining the prognosis of tumors and for discovering diagnostic genomic classifiers, but also for the identification of regulated genes and signaling pathways. Therefore, we decided to take this approach together with a highly effective C/EBPβ-shRNA capable of silencing the expression of C/EBPβ in ALK+ ALCL cells to generate a reliable signature of genes regulated by C/EBPβ. Of the original 114 genes identified by GEP, 23 of 26 selected candidate genes were confirmed by RT-qPCR to be regulated by C/EBPβ. To further validate our data, we compared our C/EBPβ-signature with published ALK-signatures. Not surprisingly, only a limited number of overlapping genes among these studies were found (*TXNIP, AQP9, ELAC2, UGCG, UBE2H, BCL2A1, DDX21* and *CCL20, G0S2, S100A9, UPK1B*) [29]–[31]. Three of the tightly regulated C/EBPβ candidate genes (*BCL2A1, DDX21 and CCL20*) were also identified in the ALK-signature [29]. Moreover, a comparative study of ALK+ ALCL to other T-NHLs also had demonstrated that some of our candidate genes (*G0S2, S100A9, UPK1B*) are differentially expressed in ALK+ ALCL tumors [31]. The overlapping detection of these genes in the different signatures indicates that at least some of them are most probably important mediators of NPM-ALK oncogenesis. Furthermore, our results underscore the relevance and contribution of C/EBPβ transcriptional activity to the expression signature of ALK+ ALCL cells.

In order to understand better the role of C/EBPβ in ALK+ ALCL pathogenesis, genes regulated by C/EBPβ-knockdown were further characterized by a Gene Ontology term enrichment analysis. Significantly overrepresented terms were related to the immune system process (29%), cell proliferation (21%), apoptosis (21%) and inflammatory response (13%), which is in agreement with the well-known functions of C/EBPβ in macrophage differentiation, inflammatory response and fat metabolism [2]. Of the 114 candidate genes that composed the C/EBPβ signature, 26 genes were selected for further analysis based on its high mRNA expression and/or strong regulation by C/EBPβ, possible pathway connections and potential promoter binding sites for C/EBPβ.

To confirm potential direct transcriptional targets of C/EBPβ, ChIP analysis was performed in 12 of the 23 validated candidate genes. Promoter binding of C/EBPβ was detected in six of the investigated genes, which included *BCL2A1, G0S2, TRIB1, S100A9, DDIT4, and DDX21.* The antiapoptotic gene *BCL2A1* is of particular interest because it is a member of the Bcl-2 protein family largely restricted to the hematopoietic compartment, and specifically a direct target of pre TCR-signalling, thereby, promoting the viability of T-cells. *BCL2A1* is a direct transcription target of NF-kappa B (NF-κB) in response to inflammatory stimuli and is upregulated by different extracellular signals such as B-cell receptor stimulation, and CD40 signaling [32]. BCL2A1 protects cells from various death stimuli including TNFα stimulation, p53 activation, T- and B-cell receptor triggering and IL3 deprivation, indicating a cytoprotective function essential for lymphocyte activation, as well as survival [32]. Importantly, BCL2A1 was described together with C/EBPβ as critical target of ALK-activity, necessary for sustaining the survival and/or growth of ALK+ ALCL cells [29]. Our results corroborate and extend these previous findings establishing *BCL2A1* as a direct transcription target of C/EBPβ necessary to promote survival of ALK+ ALCL cells. We confirmed the antiapoptotic key role of BCL2A1 in ALK+ ALCL cells, which showed significant growth retardation (60%) and cell death (34.1%), 48 hours after BCL2A1 silencing. Of note, no difference in BCL2A1 protein expression was found in ALK+ vs. ALK- ALCL primary cases. Accordingly, BCL2A1, in addition to being a transcriptional target of C/EBPβ, can also be regulated by other transcription factors such as NF-κB or AP1 [33]. Interestingly, it has been shown that NF-κB activity is either very low or not present in ALK+ ALCL and that its activity is blocked directly by NPM-ALK [34]. In contrast, NF-κB is constitutively expressed in many peripheral T-cell lymphomas and in classical Hodgkin lymphoma [35], [36]. NF-κB activation has been related to oncogenesis through several mechanisms, mainly promoting cellular proliferation and inhibition of apoptosis [37]. Therefore, it is tempting to speculate that NF-κB might be one alternative pathway responsible for BCL2A1 induction in ALK- ALCL tumors. Regardless of the mechanism, the universal expression of BCL2A1 implies that this protein is necessary for survival in both ALK+ and ALK- ALCL, and therefore, it might be a potential candidate for targeted therapy.

*G0S2* is a gene differentially expressed in primary ALK+ ALCL with very high mRNA and protein expression levels and is tightly regulated by C/EBPβ, but whose biological functions are not clearly understood. G0S2 is widely expressed in normal human tissues and is present at particularly high levels in peripheral blood [38]. G0S2 was originally reported to have a role in the re-entry of cells from G0 into the G1 phase of the cell cycle, and therefore, involved in promoting cell proliferation [39]. Accordingly, the inhibition of G0S2 resulted in cell arrest in endometrial cells [40]. However, a recent study suggested that the upregulation of G0S2 was associated with cell-cycle withdrawal rather than cell proliferation [41]. Moreover, ectopic expression of G0S2 was shown to induce apoptosis in diverse human cancer cell lines in which endogenous *G0S2* was normally epigenetically silenced [38]. Although these results seem contradictory, it might also indicate that G0S2 has a dual role, on one side promoting cellular proliferation and on the other side inducing apoptosis under certain circumstances. Our results in ALK+ ALCL support a role of G0S2 mainly in cell cycle progression with a minor role in apoptosis, potentially contributing to ALK-mediated oncogenesis.

Due to the emerging role of C/EBPβ in the control of ribosomal RNA (rRNA) biogenesis during adipogenesis [42], it was remarkable to find that *DDX21*, previously known as RNA helicase II/Guα is a downstream target of C/EBPβ. DDX21 (DEAD box polypeptide 21) belongs to the DExD/H box family of proteins that play important roles in basically all aspects of RNA metabolism [43]. Members of this family have been shown to act as RNA helicases with RNA unwinding and foldase activity, and are involved in the production of ribosomal RNA (rRNA). Interestingly, several DExD/H box proteins have been shown to be multifunctional and in addition to act as RNA helicases in processes such as pre-mRNA processing, translational initiation and ribosome assembly, play important roles in the regulation of gene transcription. DDX21 is a nucleolar protein with RNA helicase and folding activity involved in the regulation of rRNA biogenesis, it is critical for 18S and 28S rRNA biosynthesis and maintenance [27], [28], [44]. Loss of DDX21 results in blocking of rRNA biogenesis, slow metabolism and reduces cell proliferation [25], [28]. In contrast high levels of DDX21 expression were shown to be associated with low survival and rapid relapse in breast cancer [45]. In previous studies, DDX21 was identified as a functional interacting partner for JUN in human cells [46], supporting JUN mediated target gene activation, whereas JUN regulated the rRNA binding and nucleolar localization of DDX21 [26]. The function of both proteins seems to be necessary to ensure cell growth. Accordingly, knockdown of *DDX21* in SUDHL1 cells resulted in blocking of rRNA biogenesis, and a 65% reduction in cell proliferation compared to control cells. Annexin V/propidium iodide stainings after DDX21 knockdown indicated that *DDX21* is also essential for survival of ALK+ ALCL cells. Production of rRNA is tightly coordinated with cell growth and proliferation, and reduced rRNA production after DDX21 silencing seems to result in decreased protein synthesis, slow metabolism, and inhibition of cell growth and proliferation.

**Figure 7:**
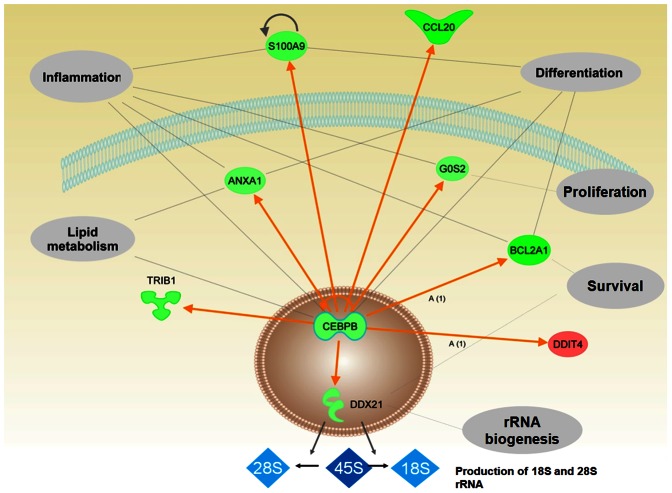
Pathway analysis of genes regulated by C/EBPβ. Gene-gene connections (orange arrows) were analyzed by Ingenuity Pathway software and associated biological functions are shown. Genes depicted in green (red) are down (up)-regulated upon C/EBPβ knockdown in our microarray dataset. Shown are the nucleus, intra- and extracellular space and part of the cell membrane (Figure was designed with Ingenuity IPA software).

In summary, using GEP analysis before and after C/EBPβ silencing in ALK+ ALCL cells, we were able to identify several direct downstream targets of C/EBPβ that might contribute to the ALK-mediated lymphoid transformation. Using the Ingenuity pathway tool, we constructed a model (Figure 7) to illustrate the important role of C/EBPβ in different cellular pathways and its most important downstream targets.

## Supporting Information

Figure S1 **Lentiviral transduction of C/EBPβ shRNA in SUDHL-1 cell line and gene expression profiling.** (**A**) RT-qPCR analysis of *C/EBPβ* mRNA in the transduced KiJK cells three days after infection. Values were normalized to *TBP* and data were analyzed according to the 2^−ΔΔCp^ method. Results are depicted as mRNA amount relative to untreated SUDHL-1 cells. Error bars indicate SD (n = 4). (**B)** Western Blot analysis of the different C/EBPβ isoforms (liver-enriched activation protein (LAP*, LAP), liver-enriched inhibitory protein (LIP) in the transduced KiJK cells three days after infection demonstrates successful knockdown. Each lane contains 20 µg protein extract. α-Tubulin was used as loading control. (**C**) Proliferation curves of the controls and C/EBPβ-shRNA infected KiJK cells are depicted up to 7 days after infection. Error bars indicate SD (n = 3).
(TIF)

Figure S2 **Lentiviral transduction of DDX21 shRNA in Mac-2A and Granta 519 cells.** RT-qPCR analysis, Western Blot analysis and proliferation curves of the controls and DDX21-shRNA infected cells. (**A**) Mac-2A. (**B**) Granta 519. RT-qPCR *DDX21* mRNA values three days after infection were normalized to *TBP* and data were analyzed according to the 2^−ΔΔCp^ method. Results are depicted as mRNA amount relative to untreated cells (upper part). Western Blot analysis of the different C/EBPβ isoforms (liver-enriched activation protein (LAP*, LAP), liver-enriched inhibitory protein (LIP) in the transduced cells three days after infection demonstrates successful knockdown. Lanes contain A: 5,5 µg, B: 20 µg protein extract. α-Tubulin was used as loading control (middle part). Proliferation curves of the controls and DDX21-shRNA infected cells are depicted up to the indicated time points after infection. Error bars indicate SD (n = 3) (lower part).
(TIF)

File S1 **Table S1, S2, S3, S4.** Table S1. Indicated primers and probes were combined using the Universal ProbeLibrary System for validation of gene expression regulation by C/EBPβ of the 26 candidate genes by RT-qPCR. All primers were designed for intron-spanning multiplex assays with TBP. * intron-spanning not possible. Table S1: Indicated primers and probes were combined using the Universal ProbeLibrary System for validation of gene expression regulation by C/EBPβ of the 26 candidate genes by RT-qPCR. All primers were designed for intron-spanning multiplex assays with TBP. * intron-spanning not possible. Table S2: Primer sequences to amplify 49 promoter sequences of 11 genes with potential C/EBPβ binding sites applying the QuantiTect Sybr Green PCR Kit. A BCL2A1 promoter sequence without potential C/EBPβ binding site was amplified as negative control (BCL2A1_neg). Table S3: Shown are the 169 significant differentially expressed probe sets (FDR<10%; corresponding to 114 genes) regulated upon C/EBPβ knockdown in the microarray analysis. Linear ratios are indicated (knockdown versus control). Table S4: Selected significantly (p<0.01) enriched GO terms and pathways associated with the set of 114 genes regulated upon C/EBPβ inhibition.
(DOC)
