# Supplementary figures and images for: Identification of C/EBPβ Target Genes in ALK+ Anaplastic Large Cell Lymphoma (ALCL) by Gene Expression Profiling and Chromatin Immunoprecipitation
Source: PLoS One. 2013 May 31;8(5):e64544. doi: 10.1371/journal.pone.0064544 (PMC3669320; doi:10.1371/journal.pone.0064544)

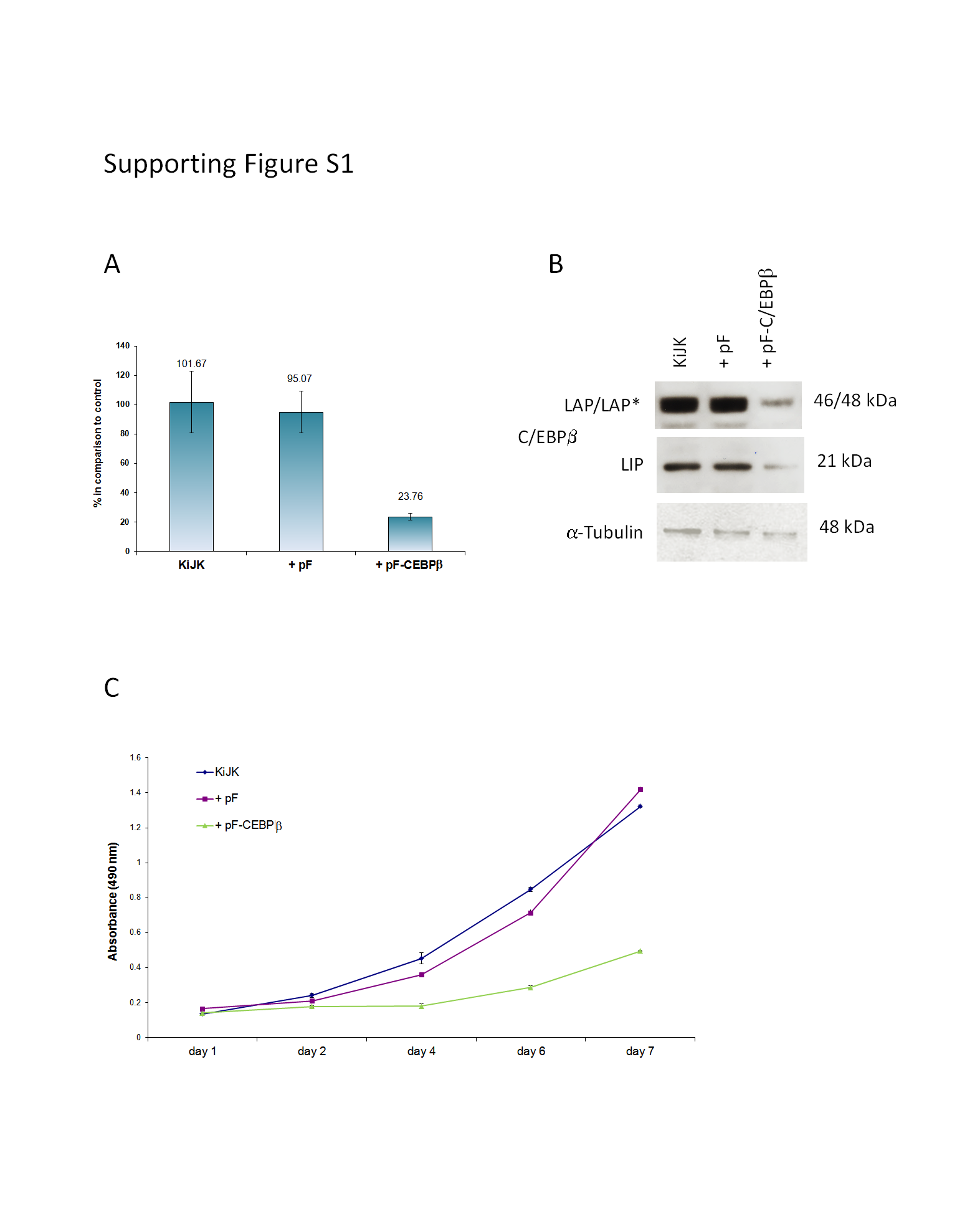

Supplement: Figure S1 — Lentiviral transduction of C/EBPβ shRNA in SUDHL-1 cell line and gene expression profiling. (A) RT-qPCR analysis of C/EBPβ mRNA in the transduced KiJK cells three days after infection. Values were normalized to TBP and data were analyzed according to the 2−ΔΔCp method. Results are depicted as mRNA amount relative to untreated SUDHL-1 cells. Error bars indicate SD (n = 4). (B) Western Blot analysis of the different C/EBPβ isoforms (liver-enriched activation protein (LAP*, LAP), liver-enriched inhibitory protein (LIP) in the transduced KiJK cells three days after infection demonstrates successful knockdown. Each lane contains 20 µg protein extract. α-Tubulin was used as loading control. (C) Proliferation curves of the controls and C/EBPβ-shRNA infected KiJK cells are depicted up to 7 days after infection. Error bars indicate SD (n = 3). (TIF) [file pone.0064544.s001.tif]

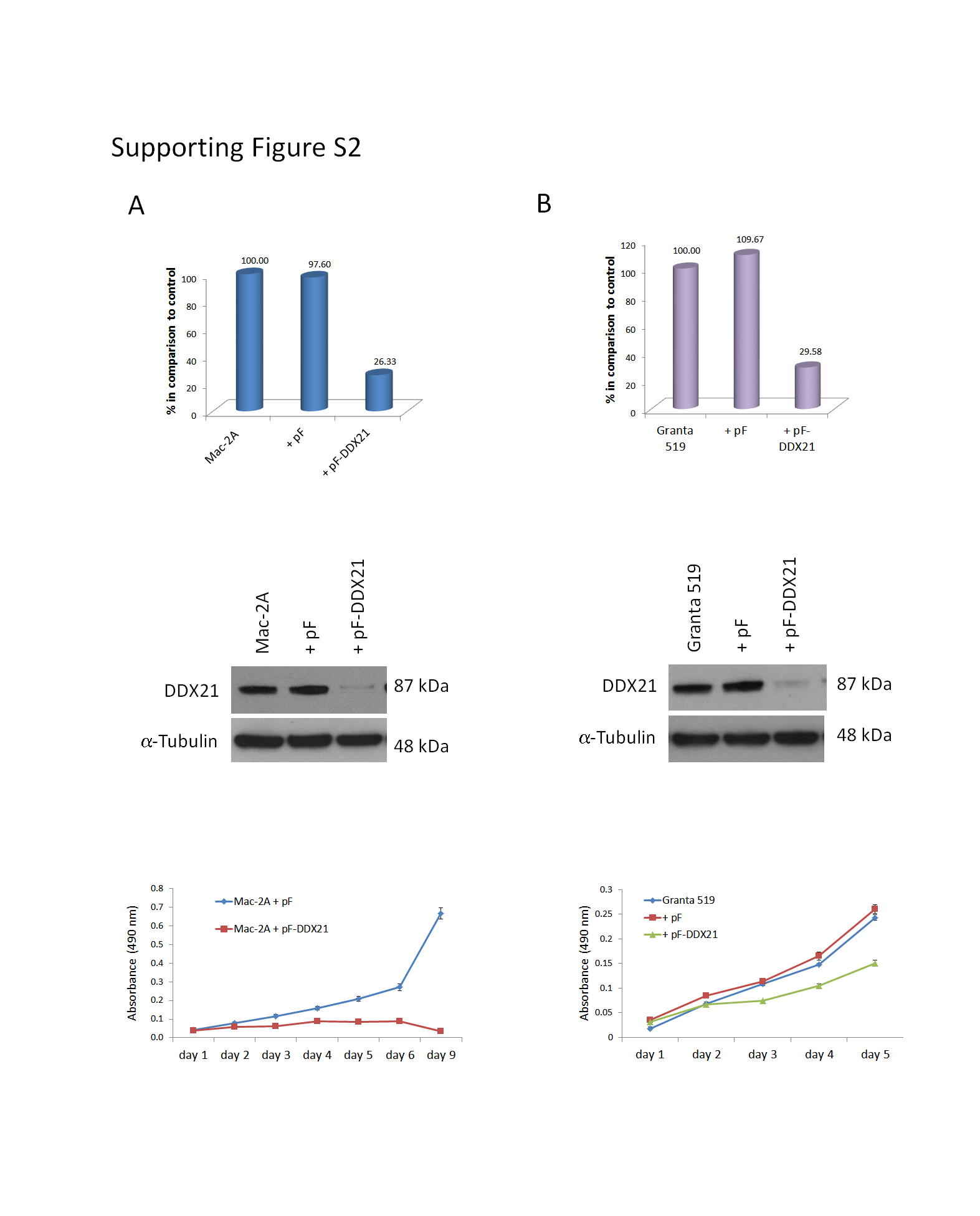

Supplement: Figure S2 — Lentiviral transduction of DDX21 shRNA in Mac-2A and Granta 519 cells. RT-qPCR analysis, Western Blot analysis and proliferation curves of the controls and DDX21-shRNA infected cells. (A) Mac-2A. (B) Granta 519. RT-qPCR DDX21 mRNA values three days after infection were normalized to TBP and data were analyzed according to the 2−ΔΔCp method. Results are depicted as mRNA amount relative to untreated cells (upper part). Western Blot analysis of the different C/EBPβ isoforms (liver-enriched activation protein (LAP*, LAP), liver-enriched inhibitory protein (LIP) in the transduced cells three days after infection demonstrates successful knockdown. Lanes contain A: 5,5 µg, B: 20 µg protein extract. α-Tubulin was used as loading control (middle part). Proliferation curves of the controls and DDX21-shRNA infected cells are depicted up to the indicated time points after infection. Error bars indicate SD (n = 3) (lower part). (TIF) [file pone.0064544.s002.tif]
