# Supplementary material for: Identification of C/EBPβ Target Genes in ALK+ Anaplastic Large Cell Lymphoma (ALCL) by Gene Expression Profiling and Chromatin Immunoprecipitation
Source: PLoS One. 2013 May 31;8(5):e64544. doi: 10.1371/journal.pone.0064544 (PMC3669320; doi:10.1371/journal.pone.0064544)
Supplement: File S1 — Table S1, S2, S3, S4. Table S1. Indicated primers and probes were combined using the Universal ProbeLibrary System for validation of gene expression regulation by C/EBPβ of the 26 candidate genes by RT-qPCR. All primers were designed for intron-spanning multiplex assays with TBP. * intron-spanning not possible. Table S1: Indicated primers and probes were combined using the Universal ProbeLibrary System for validation of gene expression regulation by C/EBPβ of the 26 candidate genes by RT-qPCR. All primers were designed for intron-spanning multiplex assays with TBP. * intron-spanning not possible. Table S2: Primer sequences to amplify 49 promoter sequences of 11 genes with potential C/EBPβ binding sites applying the QuantiTect Sybr Green PCR Kit. A BCL2A1 promoter sequence without potential C/EBPβ binding site was amplified as negative control (BCL2A1_neg). Table S3: Shown are the 169 significant differentially expressed probe sets (FDR<10%; corresponding to 114 genes) regulated upon C/EBPβ knockdown in the microarray analysis. Linear ratios are indicated (knockdown versus control). Table S4: Selected significantly (p<0.01) enriched GO terms and pathways associated with the set of 114 genes regulated upon C/EBPβ inhibition. (DOC) [file pone.0064544.s003.doc]

# Supporting Information

Supporting Methods

Cloning of C/EBPβ isoforms into the lentiviral pRRL.PPT.SF.i2GFPp vector and virus production

Primers were designed to amplify LAP* and LAP from healthy donors blood and modified for cloning into the BamHI site of the vector using the In-Fusion primer designer (LAP* forward 5’-TACCGTCGACGGATCCATGCAACGCCTGGTGGCC-3’, LAP forward 5’- TACCGTCGACGGATCCATGGAAGTGGCCAACTTCTACTAC-3’, reverse 5’-GGAGAGGGGCGGATCCCTAGCAGTGGCCGGAGGA-3’). PCR products were generated using Phusion™ Hot Start High-Fidelity DNA Polymerase (Thermo Fisher Scientific) with the stated conditions of the manual and the addition of 2% DMSO. PCR products were cloned into pRRL.PPT.SF.i2GFPp according to the manufacturer’s protocol (Clontech Laboratories, Mountain View, CA, USA). Production of virus containing the pRRL.PPT.SF.i2GFPp vectors was performed transfecting 3x10^6^ cells in 10 cm plates using 30 µl TransIT-2020, 2 µg pHCMV-G, 6 µg pCMVdeltaR8.9 and 6 µg of the respective pRRL.PPT.SF.i2GFPp derivate according to the manufacturer’s manual (Mirus Bio, Madison, MI, USA).

Luciferase Reporter Assay – Cloning and Transfection

The regulatory site of the intron region of *DDX21* (DDX21_I1 (Table S2 in File S1)) was cloned into the reporter plasmid pRR-High (Active Motif, Carlsbad, CA, USA). Primers from ChIP experiments (S2) were modified for cloning between the BstXI and ApaI site of the vector using the In-Fusion primer designer. PCR products were generated using AmpliTaq Gold® DNA Polymerase (Applied Biosystems) with DNA from healthy donors blood according to the manual. PCR products were cloned into pRR-High according to the manufacturer’s protocol (Clontech Laboratories). 5x10^5^ HEK293T cells were transiently cotransfected after 24 hours with 1.5 µg of the pRR-High-DDX21_I1 reporter construct and 1 µg of pRRL.PPT.SF.i2GFPp vector containing the C/EBPβ  isoforms LAP* and LAP (section “Virus production”) in 6-well plates using 5 µl TransIT-2020, according to the manufacturer’s manual (Mirus Bio). The RapidReporter® Gaussia Luciferase Assay was performed according to the manufacturer’s instructions (Active Motif). DDX21 reporter activities were normalized to Gaussia luciferase activity and *t*-test was used to determine statistical significance of results.

Supporting Tables

Table S1: Primers and probes used in RT-qPCRs (Universal ProbeLibrary System)

| **Genes** | **5'-Primer sequence-3'** | **Probe** |
| --- | --- | --- |
| DDX21_F | AGCTGGGTGTTTGCTTTGAT | 2 |
| DDX21_R | CAGCGTCGTGAATCATGC |  |
| S100A10_F | GAGTTCCCTGGATTTTTGGAA | 76 |
| S100A10_R | CACTGGTCCAGGTCCTTCAT |  |
| G0S2_F | GAGAGGAGGAGAACGCTGAG | 15 |
| G0S2_R | GCGGGAATGACCTTAGTGG | * |
| UPK1B_F | GTCACCAAAACCTGGGACAG | 1 |
| UPK1B_R | TCCGGAAGGCAGATGTGTAT |  |
| CPD_F | AACAAATGGAGCTAGTTGGTATAATG | 79 |
| CPD_R | CAATTTGTTTGTAAATAGTTCCAGTCC |  |
| PLA2G4A_F | TGAATCACACGAACCCAAAG | 11 |
| PLA2G4A_R | CGATGAATCCAACTTGCTTG |  |
| BCL2A1_F | CAGGAGAATGGATAAGGCAAA | 75 |
| BCL2A1_R | CCAGCCAGATTTAGGTTCAAA |  |
| TM4SF1_F | TTTCTGGCATCGTAGGAGGT | 5 |
| TM4SF1_R | GTTTGCCACAGTTTTCATGG |  |
| CDK5RAP2_F | CCCCTTCACTGTGAGCAGA | 8 |
| CDK5RAP2_R | TGGTGTTTTGCAACTTCTTTTC |  |
| NCOA7_F | TCAGTGAGCCAACAACCAAG | 14 |
| NCOA7_R | GCTGCATGTGCTCTTCCTG |  |
| GLUL_F | TACCCTCATGGGGACAGATG | 57 |
| GLUL_R | TGCTCCCACACCACAGTAAT |  |
| JUN_F | CCAAAGGATAGTGCGATGTTT | 19 |
| JUN_R | CTGTCCCTCTCCACTGCAAC | * |
| S100A9_F | GTGCGAAAAGATCTGCAAAA | 85 |
| S100A9_R | TCAGCTGCTTGTCTGCATTT |  |
| PDZD2_F | TTGCTGTCCAGAAAGACCATC | 1 |
| PDZD2_R | GCATCGTGTACAGCCACACT |  |
| TRIB1_F | CGGCTCTTCAAGCAGATTGT | 87 |
| TRIB1_R | GACTTTCTAGTCTAAGCTGGGTTCTC |  |
| CDKN2A_F | GTGGACCTGGCTGAGGAG | 34 |
| CDKN2A_R | CTTTCAATCGGGGATGTCTG |  |
| DDIT4_F | CTGGACAGCAGCAACAGTG | 69 |
| DDIT4_R | ACACCCCATCCAGGTAAGC |  |
| IL6ST_F | ATATTGCCCAGTGGTCACCT | 25 |
| IL6ST_R | TTTGCTTCTATTTCCACAACACTT |  |
| ALB_F | CAAAGATGACAACCCAAACCTC | 54 |
| ALB_R | GGATGTCTTCTGGCAATTTCA |  |
| PTPRC_F | TGATTCAGGTCGTCAAACAAA | 60 |
| PTPRC_R | CGTTTGCTGAGATCCATCC |  |
| KLF6_F | GATGAGTTAACCAGGCACTTCC | 85 |
| KLF6_R | AGAGGTGCCTCTTCATGTGC |  |
| ANXA1_F | CAGTAAGCATGACATGAACAAAGTT | 85 |
| ANXA1_R | GAAAGCTGGTTTGCTTGTGG |  |
| S100A8_F | CAAGTCCGTGGGCATCAT | 78 |
| S100A8_R | GACGTCGATGATAGAGTTCAAGG | * |
| CCL20_F | GCTGCTTTGATGTCAGTGCT | 39 |
| CCL20_R | TCAAAGTTGCTTGCTGCTTC |  |
| STAT4_F | TTTATCCCCATCTCAACAATCC | 21 |
| STAT4_R | TGGGAAGAAGGTCTGATGGA |  |
| BIRC3_F | AGGGGCAGATGGCTCTGTA | 29 |
| BIRC3_R | TGTCAGCTGTGACCCTTATGA | * |

Table S2: Primers ChIP assay

| **Promoter regions** | **5'-Forward primer-3'** | **5'-Reverse primer-3'** |
| --- | --- | --- |
| BCL2A1_P1 | GCTGCAAAACATTTTCTTTTCTT | CGGGAGACAGAAGTTGCAGT |
| BCL2A1_P2 | GGCTGGTCTCAAACTCCTGA | TGGGTAGAAGGTTTTCTTTTGTG |
| BCL2A1_P3 | TCAGTGGCATTTAGTACCTTCG | GAGCGAGACTCTGTCTCCAAA |
| BCL2A1_P4 | TGAGGAAGTGGCTTCTCTGAA | TCCTTCCTCCTAGTCACTTTCG |
| CDKN2A_P1 | TTACACGCAGGAGGGGAAG | CCCTCAACCCTTGATTTTCA |
| CDKN2A_P2 | AGGAAAGAGAAATGTGAGAAGTGTG | GTCATTGGAAGGACGGACTC |
| CDKN2A_P3 | TTTGAGTCGGAGTCTCATTCTG | AAATTAGCTGGCCATGGTG |
| CDKN2A_PB1 | GCGTGCAGCGGTTTAGTTTA | AGGAAAGCGAGGTCATCTCA |
| CDKN2A_PB2 | TGGTCTTTGGATCACTGTGC | TAATACGGACGGGGGAGAAT |
| Jun_P1 | CTCGATGCTTCTCACAGGAA | CTGCCTCGCAGTTTCCAT |
| Jun_P2 | CGGGCCCAGAGAAGAATC | CCCCTAAAAATAGCCCATGA |
| PTPRC_P1 | ACAGCAAGCTCACAGAACACA | TAGGCCTAATAAAACATCAGTGTCA |
| PTPRC_P2 | CCACAAGGGCTCTGTCTGTC | TGGCAAAAAGTATAAATTCAACAA |
| PTPRC_PB1 | GCTGAGTCATGGGTATAAGGGTA | CACGTTTGACTATATGCATTTTATAGC |
| PTPRC_PB2 | GGGAAGCCTGAATTGTGAAA | CACAGCATGAGCTCAAAGGTT |
| PTPRC_PB3 | CCTCCTGTGCCAACCTTTTA | AGTTTGGACAGCCCAAGAAG |
| PTPRC_PB4 | TCTGTAGAATGCATGTGTCGTG | TGGGACATCTGCAATCAGAA |
| S100A9_P1 | GAGCTCTTCCCAACTTTCCA | GGCAGTTTACTTAACTTCTCTGTGC |
| S100A9_P2 | GATGCTGAGAGATGCTAGAAACA | GGACCTGAGCAAGGTCACATA |
| S100A9_P3a | CAGGTGCATCTCAGCAGTGT | CCCCTCCTGACTGCTTTGT |
| S100A9_P3b | CATTCCAAGAGGGCTTTGTG | GTGGGCAAGATGTGTCCTTC |
| S100A9_P3c | CAAGTGCCCCAGTCAGGA | AGTTGGGTCAGGGAAGGAAA |
| S100A9_PB1 | TTCCTCCCAAACCAGTTTCA | CGGAAGATTGCTTCACAGGT |
| S100A9_PB3 | TCCCAACTCTTGGTTTTCCA | TTGGTGGAAGGTGTTGATGA |
| TM4SF1_PB1 | AAATCCATCCTGCAGAGCTT | TTTACAACCTGGGGCAAATC |
| TM4SF1_PB2 | TGGTATATTCTGCATTTTACAAGTGG | TGAGGATGTAACATGGGAGGA |
| TM4SF1_PB3-1 | TCCTAGATTGGGGTCAAAAA | TGTGTAAGTTTCTTCCACTGCAT |
| TM4SF1_PB3-2 | TACCTCCACATGGTCCGAGT | AATTCCACCAAGAGCCAAGA |
| TM4SF1 | CCCCTCCTTCTGCAAGGTA | TTACCCAGCCAGATCCTGAG |
| DDIT4 | CAACAAGCGACAGAGCTTGA | TGGGAGACCCACTTATGGAG |
| DDIT4_PB1-1 | CATTCTAATCTGGGTGACAGAGTG | TGAGGAGTCTTTGCAAATAAGG |
| DDIT4_PB1-2 | GAGGCCCTCACTTCAGCTTT | GATGAGAAGCCAGCAGAAGC |
| TRIB1_P1 | GGCCTGTGAGTGTGTGTGTA | GTGTATGAGAGCGAGCGAGA |
| TRIB1_P2 | GCCAGGCTGGTCTCAAACT | GGACTTAGGCCAAGCACAGT |
| TRIB1_P3 | ATCTGGGAGCTCTGAAGCTG | TTGCCTTGACTGAGAAGAGC |
| TRIB1_P4 | CGCCTGTAATCCCAGCTACT | CGCCTAAGCTAGAGTGCAGTG |
| TRIB1_PB1 | CCCCAAAACCTCAGAAACCT | TCATCCCAACAGAGAAGAGGA |
| TRIB_PB2-1 | TCGAATGGGAGAAAAAGCAA | TCAAAGATGGCAGATTTATTGG |
| TRIB_PB2-2 | GACTGCTACGGCAGCTTCTC | TCCAAAAACGGAGCAGAGTC |
| DDX21 | TCAGGCTCCTGAGTCTGGTC | GCTTCTAGTTGCTTTTGAGTGG |
| DDX21_P1alt | TTGATTGTGCTCTGTGTGTCA | TCTGTTATTTTTGGAAACATTACCTT |
| DDX21_P1-2 | CGGAGGTGGGAAGATCACT | CCCTGCCTGGCTAATTTT |
| DDX21_P2 | GAAGGATGAGAATCGCTGGA | TGAAAATCGGCCTTTTTGTT |
| DDX21_P2-2 | GGCCGATTTTCAATGTCAGA | CGACACAGCAAAACCCCTAT |
| DDX21_P3 | CGTTTCCAAGAGATGATTAAGGA | TAGCCCCAGCCTGATAAATG |
| DDX21_I1 | GCGGCAGATTTGTACTCAGG | CATGTACCTCTGGATTTGAGCA |
| G0S2_P1 | CTGGACCAGTCAATTTCAGAAT | TGATTAGTGAGCTTTCCCAATATC |
| G0S2_P2 | TTCAGACCCCCAAAGAAAGA | CTGGCCAGAAAATTGCAAAG |
| G0S2_P3 | AATAAAACCAACCAGGCAGGA | TATTCGGAAGGGTTGTCACC |
| CCL20 | TGACTGGTTCTGGAAAGCAA | TCTGAAAATAGAGGATTAACAGCGATA |
| BCL2A1_neg | GCAGTTCCTTTGACTGCCTA | CCGTATTAGGCGCTGAAGTT |

Table S3: Significantly regulated genes after C/EBPβ knockdown

| **Probe** | **Gene symbol** | **Representative Public ID** | **SUDHL-1 RMA ratio BH<0.1** | **KiJK RMA ratio BH<0.1** |
| --- | --- | --- | --- | --- |
| 243483_at | TRPM8 | AI272941 | 5.84 | 1.72 |
| 217127_at | CTH | AL354872 | 5.00 | 2.26 |
| 210538_s_at | BIRC3 | U37546 | 3.88 | 2.13 |
| 211298_s_at | ALB | AF116645 | 3.61 | 1.38 |
| 1555564_a_at | CFI | BC020718 | 3.13 | 1.71 |
| 207602_at | TMPRSS11D | NM_004262 | 2.87 | 1.94 |
| 202207_at | ARL4C | BG435404 | 2.79 | 1.99 |
| 203854_at | CFI | NM_000204 | 2.77 | 1.58 |
| 207238_s_at | PTPRC | NM_002838 | 2.66 | 1.75 |
| 202748_at | GBP2 | NM_004120 | 2.61 | 1.56 |
| 201010_s_at | TXNIP | NM_006472 | 2.52 | 1.67 |
| 212588_at | PTPRC | Y00062 | 2.47 | 1.66 |
| 1555832_s_at | KLF6 | BU683415 | 2.32 | 1.75 |
| 224606_at | KLF6 | BG250721 | 2.31 | 1.73 |
| 217996_at | PHLDA1 | AA576961 | 2.31 | 1.88 |
| 221111_at | IL26 | NM_018402 | 2.30 | 1.46 |
| 201008_s_at | TXNIP | AA812232 | 2.24 | 1.67 |
| 204401_at | KCNN4 | NM_002250 | 2.22 | 1.73 |
| 201466_s_at | JUN | NM_002228 | 2.19 | 1.58 |
| 214452_at | BCAT1 | NM_005504 | 2.17 | 1.39 |
| 234989_at | TncRNA | AV699657 | 2.16 | 1.96 |
| 212587_s_at | PTPRC | AI809341 | 2.15 | 1.70 |
| 242907_at |  | BF509371 | 2.14 | 1.68 |
| 200761_s_at | ARL6IP5 | NM_006407 | 2.09 | 1.53 |
| 219026_s_at | RASAL2 | NM_004841 | 2.04 | 1.64 |
| 1553746_a_at | C12orf64 | NM_173591 | 2.02 | 1.64 |
| 206584_at | LY96 | NM_015364 | 2.01 | 1.81 |
| 208960_s_at | KLF6 | BE675435 | 1.99 | 1.70 |
| 208961_s_at | KLF6 | AB017493 | 1.96 | 1.62 |
| 230499_at |  | AA805622 | 1.94 | 1.54 |
| 222810_s_at | RASAL2 | BF435513 | 1.92 | 2.06 |
| 224567_x_at | MALAT1 | BG534952 | 1.92 | 1.64 |
| 1553798_a_at | FBXL13 | NM_145032 | 1.90 | 1.47 |
| 227036_at |  | N66622 | 1.88 | 1.69 |
| 203498_at | RCAN2 | NM_005822 | 1.88 | 1.72 |
| 214657_s_at | TncRNA | AU134977 | 1.88 | 1.82 |
| 225242_s_at | CCDC80 | AW303375 | 1.86 | 1.52 |
| 202085_at | TJP2 | NM_004817 | 1.86 | 1.56 |
| 209682_at | CBLB | U26710 | 1.84 | 1.48 |
| 224797_at | ARRDC3 | AB037797 | 1.80 | 1.42 |
| 209732_at | CLEC2B | BC005254 | 1.80 | 1.67 |
| 201009_s_at | TXNIP | AI439556 | 1.78 | 1.60 |
| 228617_at | XAF1 | AA142842 | 1.78 | 1.47 |
| 204070_at | RARRES3 | NM_004585 | 1.76 | 1.94 |
| 200985_s_at | CD59 | NM_000611 | 1.75 | 1.43 |
| 223395_at | ABI3BP | AB056106 | 1.75 | 1.66 |
| 231807_at | KIAA1217 | AL157473 | 1.74 | 1.91 |
| 204268_at | S100A2 | NM_005978 | 1.74 | 1.40 |
| 205048_s_at | PSPH | NM_003832 | 1.71 | 1.78 |
| 208561_at | ABCC9 | NM_020297 | 1.70 | 0.62 |
| 205194_at | PSPH | NM_004577 | 1.67 | 1.78 |
| 218901_at | PLSCR4 | NM_020353 | 1.64 | 1.69 |
| 213397_x_at | RNASE4 | AI761728 | 1.62 | 1.98 |
| 205552_s_at | OAS1 | NM_002534 | 1.62 | 2.05 |
| 242455_at | POU3F2 | BE855760 | 1.61 | 1.54 |
| 202208_s_at | ARL4C | BC001051 | 1.61 | 1.68 |
| 202887_s_at | DDIT4 | NM_019058 | 1.59 | 1.41 |
| 206118_at | STAT4 | NM_003151 | 1.58 | 1.40 |
| 202687_s_at | TNFSF10 | U57059 | 1.58 | 1.94 |
| 1554438_at | KIAA1217 | BC018764 | 1.57 | 1.89 |
| 233504_at | C9orf84 | AA629020 | 1.56 | 2.49 |
| 200983_x_at | CD59 | BF983379 | 1.54 | 1.41 |
| 219691_at | SAMD9 | NM_017654 | 1.53 | 2.04 |
| 242051_at |  | AI695695 | 1.52 | 1.52 |
| 202688_at | TNFSF10 | NM_003810 | 1.50 | 2.03 |
| 226603_at | SAMD9L | BE966604 | 1.50 | 1.86 |
| 228167_at | KLHL6 | AW574798 | 1.48 | 1.44 |
| 227606_s_at | STAMBPL1 | AI638611 | 1.48 | 1.44 |
| 213293_s_at | TRIM22 | AA083478 | 1.47 | 1.63 |
| 209118_s_at | TUBA1A | AF141347 | 1.46 | 1.65 |
| 228531_at | SAMD9 | AA741307 | 1.44 | 2.29 |
| 1555292_at | FAM40B | BC019064 | 1.43 | 1.35 |
| 219696_at | C1orf218 | NM_019049 | 1.43 | 1.45 |
| 225959_s_at | ZNRF1 | BF432625 | 1.42 | 1.62 |
| 230805_at |  | AA749202 | 1.42 | 1.46 |
| 221962_s_at | UBE2H | AI829920 | 1.42 | 1.44 |
| 240064_at |  | AI738675 | 1.41 | 1.71 |
| 230036_at | SAMD9L | BE669858 | 1.41 | 1.92 |
| 201028_s_at | CD99 | U82164 | 1.41 | 1.50 |
| 210102_at | LOH11CR2A | BC001234 | 1.40 | 1.53 |
| 237105_at |  | AI138283 | 1.40 | 1.38 |
| 212792_at | DPY19L1 | AB020684 | 1.40 | 1.44 |
| 234819_at |  | AE000660 | 1.38 | 2.09 |
| 204881_s_at | UGCG | NM_003358 | 1.35 | 1.51 |
| 201012_at | ANXA1 | NM_000700 | 0.75 | 0.51 |
| 228565_at | KIAA1804 | AI809005 | 0.74 | 1.49 |
| 205660_at | OASL | NM_003733 | 0.73 | 1.86 |
| 201767_s_at | ELAC2 | NM_018127 | 0.71 | 0.59 |
| 222735_at |  | AW452608 | 0.71 | 0.67 |
| 226459_at | PIK3AP1 | AW575754 | 0.70 | 0.58 |
| 200648_s_at | GLUL | NM_002065 | 0.69 | 0.61 |
| 229521_at | FLJ36031 | BE466274 | 0.69 | 0.64 |
| 227883_at | FLJ36031 | AI672172 | 0.68 | 0.62 |
| 224607_s_at | SRP68 | BG398597 | 0.68 | 0.62 |
| 225750_at |  | BE966748 | 0.68 | 0.59 |
| 224654_at | DDX21 | BG164358 | 0.68 | 0.54 |
| 223809_at | RGS18 | AF076642 | 0.68 | 0.62 |
| 234776_at | DMBX1 | AL137797 | 0.68 | 0.40 |
| 218918_at | MAN1C1 | NM_020379 | 0.68 | 0.66 |
| 200872_at | S100A10 | NM_002966 | 0.67 | 0.57 |
| 225578_at | RP11-11C5.2 | AI885466 | 0.67 | 0.46 |
| 1555728_a_at | MS4A4A | AF354928 | 0.67 | 0.67 |
| 224413_s_at | TM2D2 | AF353991 | 0.66 | 0.65 |
| 211962_s_at | ZFP36L1 | BG250310 | 0.66 | 0.60 |
| 226832_at |  | BF978778 | 0.65 | 0.66 |
| 213061_s_at | NTAN1 | AA643304 | 0.64 | 0.51 |
| 208152_s_at | DDX21 | NM_004728 | 0.64 | 0.50 |
| 209362_at | MED21 | AI688580 | 0.64 | 0.57 |
| 226121_at | DHRS13 | AI076793 | 0.64 | 0.60 |
| 218772_x_at | TMEM38B | NM_018112 | 0.64 | 0.57 |
| 214124_x_at |  | AL043487 | 0.64 | 0.60 |
| 213524_s_at | G0S2 | NM_015714 | 0.63 | 0.50 |
| 223586_at | ARNTL2 | AF256215 | 0.63 | 0.53 |
| 231795_at | STON1 | BG289281 | 0.62 | 0.27 |
| 213062_at | NTAN1 | AA643304 | 0.62 | 0.50 |
| 235331_x_at | PCGF5 | AI341142 | 0.61 | 0.60 |
| 212501_at | CEBPB | AL564683 | 0.61 | 0.51 |
| 209930_s_at | NFE2 | L13974 | 0.60 | 0.47 |
| 210592_s_at | SAT1 | M55580 | 0.59 | 0.69 |
| 209363_s_at | MED21 | U46837 | 0.58 | 0.54 |
| 218538_s_at | MRS2L | NM_020662 | 0.58 | 0.39 |
| 207039_at | CDKN2A | NM_000077 | 0.56 | 0.42 |
| 1569095_at | LOC731424 | BC016366 | 0.56 | 0.71 |
| 212522_at | PDE8A | W73272 | 0.56 | 0.59 |
| 203455_s_at | SAT1 | NM_002970 | 0.55 | 0.70 |
| 241925_x_at |  | BF207870 | 0.55 | 0.52 |
| 209194_at | CETN2 | BC005334 | 0.54 | 0.53 |
| 230003_at |  | AW779917 | 0.54 | 0.52 |
| 1559777_at |  | AK057428 | 0.54 | 0.66 |
| 201627_s_at | INSIG1 | NM_005542 | 0.54 | 1.62 |
| 241866_at | SLC16A7 | AW975728 | 0.51 | 0.60 |
| 212195_at | IL6ST | AL049265 | 0.51 | 0.65 |
| 228285_at | TDRD9 | AI989706 | 0.51 | 0.49 |
| 207057_at | SLC16A7 | NM_004731 | 0.50 | 0.57 |
| 227034_at | ANKRD57 | BE669553 | 0.49 | 0.62 |
| 219496_at | ANKRD57 | NM_023016 | 0.49 | 0.70 |
| 209493_at | PDZD2 | AF338650 | 0.48 | 0.59 |
| 211965_at | ZFP36L1 | BE620915 | 0.48 | 0.47 |
| 201626_at | INSIG1 | BG292233 | 0.47 | 1.58 |
| 201940_at | CPD | AA897514 | 0.46 | 0.52 |
| 201943_s_at | CPD | NM_001304 | 0.45 | 0.55 |
| 210065_s_at | UPK1B | AB002155 | 0.45 | 0.47 |
| 219607_s_at | MS4A4A | NM_024021 | 0.44 | 0.34 |
| 201941_at | CPD | BE349147 | 0.42 | 0.62 |
| 201942_s_at | CPD | D85390 | 0.41 | 0.58 |
| 210145_at | PLA2G4A | M68874 | 0.40 | 0.44 |
| 205681_at | BCL2A1 | NM_004049 | 0.39 | 0.29 |
| 209386_at | TM4SF1 | AI346835 | 0.39 | 0.36 |
| 210064_s_at | UPK1B | NM_006952 | 0.38 | 0.42 |
| 207275_s_at | ACSL1 | NM_001995 | 0.38 | 0.37 |
| 220935_s_at | CDK5RAP2 | NM_018249 | 0.37 | 0.49 |
| 225344_at | NCOA7 | AL035689 | 0.37 | 0.53 |
| 233540_s_at | CDK5RAP2 | AK025867 | 0.37 | 0.46 |
| 1555431_a_at | IL31RA | AF106913 | 0.37 | 0.67 |
| 201963_at | ACSL1 | NM_021122 | 0.37 | 0.32 |
| 215034_s_at | TM4SF1 | AI189753 | 0.31 | 0.34 |
| 209387_s_at | TM4SF1 | M90657 | 0.31 | 0.34 |
| 205476_at | CCL20 | NM_004591 | 0.30 | 0.27 |
| 243541_at | IL31RA | AI123586 | 0.24 | 0.58 |
| 202241_at | TRIB1 | NM_025195 | 0.23 | 0.42 |
| 223767_at | GPR84 | AF237762 | 0.19 | 0.21 |
| 203535_at | S100A9 | NM_002965 | 0.19 | 0.42 |
| 213413_at | STON1 | BG434174 | 0.17 | 0.23 |
| 229230_at | OSTalpha | AA702685 | 0.17 | 0.56 |
| 205568_at | AQP9 | NM_020980 | 0.15 | 0.30 |
| 202917_s_at | S100A8 | NM_002964 | 0.09 | 0.37 |

**Table S4: GO terms, associated with the differentially expressed genes**

| **GO-Term** | **# Genes (observed)** | **GO-Term id** | **P-value** | **Adjusted p-value** | **# Genes (ob-served)** | **# Genes (expected)** | **# Genes (total)** | **List of observed genes** | **Gene ids** |
| --- | --- | --- | --- | --- | --- | --- | --- | --- | --- |
| **Immune system process (18)** | 18 | GO:0002376 | 4.88E-05 | n/a | 18 | 6.35375202 | 982 | AQP9, IL6ST, IL31RA, GBP2, CBLB, TNFSF10, LY96, TRIM22, OAS1, ABCC9, S100A9, CCL20, CFI, OASL, KLF6, PTPRC, CEBPB, CDKN2A | 366, 3572, 133396, 2634, 868, 8743, 23643, 10346, 4938, 10060, 6280, 6364, 3426, 8638, 1316, 5788, 1051, 1029 |
| **Apoptosis (13)** | 13 | GO:0006915 | 4.97E-03 | n/a | 13 | 5.80378367 | 897 | PHLDA1, IL6ST, IL31RA, BIRC3, TNFSF10, DDIT4, XAF1, ANXA1, PTPRC, CEBPB, ALB, BCL2A1, CDKN2A | 22822, 3572, 133396, 330, 8743, 54541, 54739, 301, 5788, 1051, 213, 597, 1029 |
| **Cell proliferation (13)** | 13 | GO:0008283 | 5.97E-03 | n/a | 13 | 5.93318799 | 917 | PLA2G4A, BCAT1, IL6ST, IL31RA, CTH, INSIG1, RARRES3, POU3F2, TRIB1, JUN, ANXA1, PTPRC, CDKN2A | 5321, 586, 3572, 133396, 1491, 3638, 5920, 5454, 10221, 3725, 301, 5788, 1029 |
| **Inflammatory response (8)** | 8 | GO:0006954 | 1.13E-03 | n/a | 8 | 2.08340952 | 322 | S100A8, IL6ST, LY96, S100A9, CCL20, CFI, ANXA1, CEBPB | 6279, 3572, 23643, 6280, 6364, 3426, 301, 1051 |
| **C/EBP beta pathway (6)** | 6 | PW_CEBPB_HOMO_ SAPIENS | 7.16E-03 | n/a | 6 | 1.71748688 | 173 | IL6ST, TRIB1, JUN, STAT4, CEBPB, ALB | 3572, 10221, 3725, 6775, 1051, 213 |
| **Cytokine-mediated signaling pathway (4)** | 4 | GO:0019221 | 8.71E-04 | n/a | 4 | 0.42703425 | 66 | IL6ST, IL31RA, KLF6, PTPRC | 3572, 133396, 1316, 5788 |
